# Optimal sampling and statistical inferences for Kumaraswamy distribution under progressive Type-II censoring schemes

**DOI:** 10.1038/s41598-023-38594-9

**Published:** 2023-07-26

**Authors:** Osama E. Abo-Kasem, Ahmed R. El Saeed, Amira I. El Sayed

**Affiliations:** 1 Department of Statistics, Faculty of Commerce, Zagazig University, Zagazig, Egypt; 2 Department of Basic Sciences, Obour High Institute for Management & Informatics, Al Qalyubia, Egypt; 3 Department of Basic Sciences, Obour Higher Institute for Management and Information Technology, Al Qalyubia, Egypt

**Keywords:** Applied mathematics, Statistics

## Abstract

In this paper, we study non-Bayesian and Bayesian estimation of parameters for the Kumaraswamy distribution based on progressive Type-II censoring. First, the maximum likelihood estimates and maximum product spacings are derived. In addition, we derive the asymptotic distribution of the parameters and the asymptotic confidence intervals. Second, Bayesian estimators under symmetric and asymmetric loss functions (Squared error, linear exponential, and general entropy loss functions) are also obtained. The Lindley approximation and the Markov chain Monte Carlo method are used to derive the Bayesian estimates. Furthermore, we derive the highest posterior density credible intervals of the parameters. We further present an optimal progressive censoring scheme among different competing censoring scheme using three optimality criteria. Simulation studies are conducted to evaluate the performance of the point and interval estimators. Finally, one application of real data sets is provided to illustrate the proposed procedures.

## Introduction

The Kumaraswamy (Kum.) distribution is similar to the Beta distribution, but it has a notable advantage of having an invertible cumulative distribution function that can be expressed in a closed-form. The Kum. distribution is a flexible distribution with two shape parameters that can be applied in finite probabilities ranging from 0 to 1, such as the COVID-19 data and the monthly water capacity of Shasta Reservoir from 1975 to 2016 (see Tu and Gui^1^. Kum.^2^^,^^3^ showed that commonly used probability distribution functions like the normal, log-normal, and beta distributions do not adequately model hydrological data such as daily rainfall and stream flow. As a result, Kum. introduced a new probability density function known as the sine power probability density function.

Kum.^4^ introduced the Kum. distribution as a versatile probability density function for double-bounded random processes. This distribution is suitable for modeling various natural phenomena with lower and upper bounds, such as individual heights, test scores, atmospheric temperatures, hydrological data, and more. Additionally, the Kum. distribution can be used when scientists need to model data with finite bounds, even if they are using probability distributions with infinite bounds in their analysis. The Kum. distribution's probability density function (pdf) is described by

1 $$f\left(x\right)=\alpha \beta {x}^{\alpha -1}{\left(1-{x}^{\alpha }\right)}^{\beta -1}$$

where 0 < x < 1 and $\alpha , \beta$ are two positive shape parameters. When $\alpha$ = 1 and $\beta$ = 1, then one can obtain a Uniform distribution $U(0, 1)$ as special case of the Kum. distribution. The cumulative distribution function (cdf) of the Kum. distribution is given by

2 $$F\left(x\right)=1-{\left(1-{x}^{\alpha }\right)}^{\beta } ,0<x<1$$

**Figure 1:**
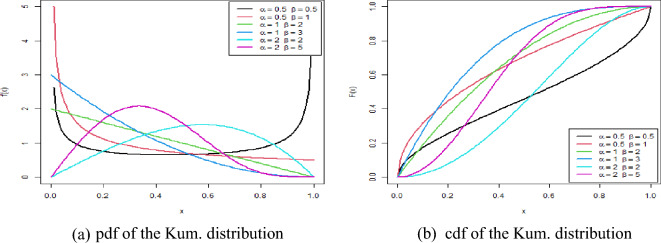
Behavior of Kum. Distribution.

Figure 1 showed the behavior of pdf and cdf of the Kum. distribution at different values of the parameters $\alpha$ and $\beta$. In Fig. 1, we notice different patterns from the distribution according to the values of the parameters, where we notice the presence of a U-shaped shape $(\alpha =0.5, \beta =0.5)$, and the gamma distribution $(\alpha =2, \beta =5)$ and we find a form that approximates the exponential distribution at the rest of the values.

In industrial life testing and medical survival analysis, it is common for the object of interest to be lost or withdrawn before failure, or for the object's lifetime to be only known within an interval. This results in a sample that is incomplete, often referred to as a censored sample. There are various reasons for removal of experimental units, such as saving them for future use, reducing total test time, or lowering associated costs. Right censoring is a technique used in life-testing experiments to handle censored samples. The conventional Type-I and Type-II censoring schemes are the most common methods of right censoring, but they do not allow for removal of units at points other than the terminal point of the experiment, limiting their flexibility. To address this limitation, a more general censoring scheme called the progressive Type-II censoring scheme (PCS-II) has been proposed. as follows:

- Suppose that $n$ unite are placed on a test at time zero with $m$ failures to be observed.
- At the first failure, say ${x}_{(1)}, {R}_{1}$ of the remaining units are randomly selected and removed.
- At the time of the second failure, ${x}_{(2)}, {R}_{2}$ of the remaining units are selected and removed.
- Finally, at the time of the ${m}^{th}$ failure the rest of the units are all removed, ${R}_{m}=n-{R}_{1}-{R}_{2}-\dots -{R}_{m-1}-m$.
- Thus, it is possible to witness the data $\left\{\left({x}_{(1)}, {R}_{1}\right),\dots ,\left({x}_{(m)}, {R}_{m}\right)\right\}$ during a gradual censorship plan. Even though ${R}_{1},{R}_{2}, \dots ,{R}_{m}$ are encompassed as a section of the data, their values are previously known.

The joint probability density function of all $m$ PCS-II order statistics is Balakrishnan and Aggarwala^5^

3 $$L\left(\alpha ,\beta \right)=\mathrm{ C}\prod_{i=1}^{m}\left(f({x}_{\left(i\right)};\alpha ,\beta )\right){\left(1-F({x}_{\left(i\right)};\alpha ,\beta )\right)}^{{R}_{i}}$$

where

$$C=n\left(n-{R}_{1}-1\right)\dots \left(n-{R}_{1}-{R}_{2}-\dots -{R}_{m}-m+1\right)$$

If ${R}_{1}={R}_{2}=\dots ={R}_{m-1}=0$, then ${R}_{i}=n-m$ which corresponds to the Type-II censoring. and If ${R}_{1}={R}_{2}=\dots ={R}_{m}=0$, then $n=m$ which corresponds to the complete sample Wu^6^.

The theory of estimation consists of these methods by which are make inference or generalization about a population parameter. Today's trend is to distinguish between the non-Bayesian and Bayesian estimate methods. Any statistical or computational strategy that does not rely on Bayesian inference is referred to as a non-Bayesian method. Bayesian method utilizes prior subjective knowledge about the probability distribution of the unknown parameters in conjunction with information provided by the sample data. Non-Bayesian method and Bayesian method of estimation will be introduced in next sections.

Gholizadeh et al.^7^ examined the performance of Bayesian and non-Bayesian estimators in estimating the shape parameter, reliability, and failure rate functions of the Kumaraswamy distribution under progressively Type-II censored samples. They used various loss functions, such as squared error loss, Precautionary loss function, and linear exponential (LINEX) loss function, to obtain the maximum likelihood and Bayes estimates of the reliability and failure rate functions, both symmetric and asymmetric. Feroze and El-Batal^8^ focused on estimating two parameters of the Kumaraswamy distribution using progressive Type-II censoring with random removals. They derived the maximum likelihood estimate for the unknown parameters and also determined the asymptotic variance–covariance matrix. Eldin et al.^9^ studied parameter estimation for the Kumaraswamy distribution using progressive Type-II censoring. They obtained estimates through both maximum likelihood and Bayesian methods. In the Bayesian approach, the two parameters were considered as random variables and estimators for the parameters were obtained by employing the squared error loss function. Erick et al.^10^ focused on estimating parameters of test units from the Kumaraswamy distribution using a progressive Type-II censoring scheme. They employed the EM algorithm to derive maximum likelihood estimates for the parameters. Additionally, they calculated the expected Fisher information matrix based on the missing value principle. EL-Sagheer's^11^ study, various methods were used to estimate unknown parameters, lifetime parameters reliability and hazard functions of a two-parameter Kumaraswamy distribution based on progressively Type-II right-censored samples. These methods included maximum likelihood, Bayes, and parametric bootstrap. The classical Bayes estimates were obtained by utilizing the Markov chain Monte Carlo technique. Sultana et al.^12^ investigated the estimation problems of unknown parameters of the Kumaraswamy distribution under Type-I progressive hybrid censoring. They derived the maximum likelihood estimates of parameters using an EM algorithm. Bayes estimates were obtained under different loss functions using the Lindley method and importance sampling procedure. Tu and Gui^1^ discussed and considered the estimation of unknown parameters featured by the Kumaraswamy distribution on the condition of a generalized progressive hybrid censoring scheme. They derived the maximum likelihood estimators and Bayesian estimators under symmetric loss functions and asymmetric loss functions, like general entropy, squared error as well as Linex loss function. Since the Bayesian estimates fail to be of explicit computation, Lindley approximation, as well as the Tierney and Kadane method, is employed to obtain the Bayesian estimates. Ghafouri and Rastogi^13^ considered the estimation of the parameters and reliability characteristics of Kumaraswamy distribution using progressive first failure censored samples. They derived the maximum likelihood estimates using an EM algorithm and compute the observed information of the parameters that can be used for constructing asymptotic confidence intervals. Also, they computed the Bayes estimates of the parameters using Lindley approximation as well as the Metropolis–Hastings algorithm. Kohansal and Bakouch^14^ conducted a study on the estimation of unknown parameters in the Kumaraswamy distribution using adaptive Type-II hybrid progressive censored samples. Firstly, they obtained the maximum likelihood estimation of the parameters using different algorithms such as Newton–Raphson (NR) method, expectation maximization (EM), and stochastic EM (SEM). They also derived the asymptotic distribution of the parameters and calculated asymptotic confidence intervals to assess the uncertainty associated with the estimates. Moreover, two bootstrap confidence intervals they achieved. Second, the Bayesian estimation of the parameters was approximated by using the Markov Chain Monte Carlo algorithm and Lindley’s method. They derived the highest posterior density credible intervals of the parameters.

This paper is organized as follows: the maximum likelihood estimators and maximum product spacing estimators of the unknown parameters of the Kum. distribution is used to create non-Bayesian estimation methods in the next section. In this part, we will look at the existence and distinctiveness of MLEs. In addition, we introduce the asymptotic distribution for the unknown parameters and generate their asymptotic confidence intervals. “Bayesian estimation methods” section focuses on obtaining the Bayes estimates of the parameters. Lindley's approximation and the Markov Chain Monte Carlo method are utilized, assuming independent gamma priors for the parameters $\alpha$ and $\beta$. Additionally, the section includes the construction of the highest posterior density credible interval for the unknown parameters. The simulation results and data analysis are presented in “Simulation study and real data analysis” section, providing an examination of the performance of the estimation methods through various simulations and real data analysis. “Optimal progressive Type-II censoring scheme” section introduces an optimal progressive censoring scheme, comparing it to different competing censoring schemes. Finally, we conclude the paper in “Conclusions” section.

## Non-Bayesian estimation methods

In this section, we examine the task of estimating Kum. parameters under PCS-II samples, employing two estimation techniques known as maximum likelihood estimators (MLEs) and maximum product spacing estimators (MPSEs).

### Maximum likelihood estimation

Suppose that $X=({x}_{\left(1\right)}, {x}_{\left(2\right)},\dots , {x}_{\left(m\right)})$ a PCS-II sample drawn from Kum. population whose pdf and cdf are given by (1) and (2), with the censoring scheme $({R}_{1},{R}_{2}, \dots ,{R}_{m})$. From (1), (2) and (3) the likelihood function is then given by:

4 $$L\left(\alpha ,\beta \right) =C{(\alpha \beta )}^{m}\prod_{i=1}^{m}{{x}_{(i)}}^{\alpha -1}{\left(1-{{x}_{(i)}}^{\alpha }\right)}^{\beta {(R}_{i}+1)-1}$$

Taking log-likelihood function of (4), $l\left(\alpha ,\beta \right)=\mathrm{log}L\left(\alpha ,\beta \right)$, one be obtaining:

5 $$l\left(\alpha ,\beta \right)=C+m\mathrm{log}\left(\alpha \right)+m\mathrm{log}\left(\beta \right)+\left(\alpha -1\right)\sum_{i=1}^{m}\mathrm{log}\left({x}_{\left(i\right)}\right)+\sum_{i=1}^{m}(\beta {(R}_{i}+1)-1)\mathrm{log}\left(1-{{x}_{\left(i\right)}}\;^{\alpha }\right)$$

Then, to estimate the parameter of the Kum. distribution, it can be obtained by finding the first partial derivative of (5) concerning parameters $\alpha$ and $\beta$ as follows:

$$ \begin{aligned} \frac{{\partial l\left( {\alpha ,\beta } \right)}}{\partial \alpha } & = { }\frac{m}{\alpha } + \mathop \sum \limits_{i = 1}^{m} {\text{log}}(x_{\left( i \right)} ) - \mathop \sum \limits_{i = 1}^{m} \left( {\beta (R_{i} + 1} \right) - 1)\frac{{x_{\left( i \right)}\;^{\alpha } {\text{log}}(x_{\left( i \right)} )}}{{\left( {1 - x_{\left( i \right)}\;^{\alpha } } \right)}} \\ \frac{{\partial l\left( {\alpha ,\beta } \right)}}{\partial \beta } & = { }\frac{m}{\beta } + \mathop \sum \limits_{i = 1}^{m} (R_{i} + 1){\text{log}}\left( {1 - x_{\left( i \right)}\;^{\alpha } } \right) \\ \end{aligned} $$

Let the partial derivative to $\alpha$ and $\beta$ respectively be 0, we have

$$ \begin{aligned} & \frac{m}{{\hat{\alpha }}} + \mathop \sum \limits_{i = 1}^{m} {\text{log}}(x_{\left( i \right)} ) - \mathop \sum \limits_{i = 1}^{m} \left( {\hat{\beta }(R_{i} + 1)} \right)\frac{{x_{\left( i \right)}\;^{{\hat{\alpha }}} {\text{log}}(x_{\left( i \right)} )}}{{\left( {1 - x_{\left( i \right)}\;^{{\hat{\alpha }}} } \right)}} = 0 \\ & \frac{m}{{\hat{\beta }}} + \mathop \sum \limits_{i = 1}^{m} (R_{i} + 1){\text{log}}\left( {1 - x_{\left( i \right)}\;^{{\hat{\alpha }}} } \right) = 0 \\ & \quad \therefore \hat{\beta } = \frac{ - m}{{\mathop \sum \nolimits_{i = 1}^{m} (R_{i} + 1){\text{log}}\left( {1 - x_{\left( i \right)}\;^{{\hat{\alpha }}} } \right)}} \\ \end{aligned} $$

The maximum likelihood estimators (MLEs) ${\widehat{\alpha }}_{MLE}$ and ${\widehat{\beta }}_{MLE}$ are the solution of the two nonlinear equations that the system needs to be solved numerically to obtain parameters estimation values.

### Maximum product spacings

Ng et al.^15^ introduced maximum product spacing (MPS) method based on PCS-II sample method, The MPS technique selects the parameter values that minimize the deviation of the observed data from a predetermined quantitative measure of uniformity.

$$G\left(\alpha ,\beta \right)=\prod_{i=1}^{m+1}\left(F({x}_{\left(i\right)};\alpha ,\beta )-F({x}_{\left(i-1\right)};\alpha ,\beta )\right)\prod_{i=1}^{m}{\left(1-F({x}_{\left(i\right)};\alpha ,\beta )\right)}^{{R}_{i}}$$

from (2), one can get:

$$G\left(\alpha ,\beta \right)=\prod_{i=1}^{m+1}\left\{{\left(1-{{x}_{(i-1)}}^{\alpha }\right)}^{\beta }-{\left(1-{{x}_{(i)}}^{\alpha }\right)}^{\beta }\right\}\prod_{i=1}^{m}{\left(1-{{x}_{(i)}}^{\alpha }\right)}^{\beta {R}_{i}}$$

The natural logarithm of the product spacings function is

$$S\left(\alpha ,\beta \right)=\sum_{i=1}^{m+1}\mathrm{log}\left\{{\left(1-{{x}_{(i-1)}}^{\alpha }\right)}^{\beta }-{\left(1-{{x}_{(i)}}^{\alpha }\right)}^{\beta }\right\}+\sum_{i=1}^{m}{\beta R}_{i}\mathrm{log}(1-{{x}_{(i)}}^{\alpha })$$

where $S\left(\alpha ,\beta \right)=\mathrm{log}G\left(\alpha ,\beta \right)$. The MPS estimators of $\alpha$ and $\beta$, denoted by ${\widehat{\alpha }}_{MPS}$ and ${\widehat{\beta }}_{MPS}$, respectively, are obtained by solving the following normal equations simultaneously

$$\frac{\partial S\left(\alpha ,\beta \right)}{\partial \alpha }=\sum_{i=1}^{m}{\beta R}_{i}\frac{-{{x}_{(i)}}^{\alpha }\mathrm{log}{(x}_{(i)})}{(1-{{x}_{(i)}}^{\alpha })} +\sum_{i=1}^{m+1}\left[\frac{\beta {(1-{{x}_{(i)}}^{\alpha })}^{\beta -1}{{x}_{(i)}}^{\alpha }\mathrm{log}{(x}_{(i)})-\beta {(1-{{x}_{(i-1)}}^{\alpha })}^{\beta -1}{{x}_{(i-1)}}^{\alpha }\mathrm{log}{(x}_{(i-1)})}{{\left(1-{{x}_{(i-1)}}^{\alpha }\right)}^{\beta }-{\left(1-{{x}_{(i)}}^{\alpha }\right)}^{\beta }}\right]=0,$$

and

$$\frac{\partial S\left(\alpha ,\beta \right)}{\partial \beta }=\sum_{i=1}^{m}{R}_{i}\mathrm{log}(1-{{x}_{(i)}}^{\alpha }) +\sum_{i=1}^{m+1}\left[\frac{{\left(1-{{x}_{(i-1)}}^{\alpha }\right)}^{\beta }\mathrm{log}\left(1-{{x}_{(i-1)}}^{\alpha }\right)-{\left(1-{{x}_{(i)}}^{\alpha }\right)}^{\beta }\mathrm{log}\left(1-{{x}_{(i)}}^{\alpha }\right)}{{\left(1-{{x}_{(i-1)}}^{\alpha }\right)}^{\beta }-{\left(1-{{x}_{(i)}}^{\alpha }\right)}^{\beta }}\right]=0$$

To obtain estimates for the parameters, the system requires solving two nonlinear equations numerically, which leads to the solution of the MPS, ${\widehat{\alpha }}_{MPS}$ and ${\widehat{\beta }}_{MPS}$.

### Asymptotic variance–covariance

The asymptotic variance–covariance matrix of the MLEs for the two parameters is the inverse of the observed Fisher information matrix as follows

$${I}^{-1}={{\left[\begin{array}{cc}\frac{{\partial }^{2}l\left(\alpha ,\beta \right)}{\partial {\alpha }^{2}}& \frac{{\partial }^{2}l\left(\alpha ,\beta \right)}{\partial \alpha \partial \beta }\\ \frac{{\partial }^{2}l\left(\alpha ,\beta \right)}{\partial \alpha \partial \beta }& \frac{{\partial }^{2}l\left(\alpha ,\beta \right)}{\partial {\beta }^{2}}\end{array}\right]}^{-1}}_{(\alpha =\widehat{\alpha },\beta =\widehat{\beta })}$$

and the variance–covariance matrix of parameters $\alpha$ and $\beta$ is given by

6 $$\therefore {I}^{-1} =\left[\begin{array}{cc}var(\widehat{\alpha })& cov(\widehat{\alpha },\widehat{\beta })\\ cov(\widehat{\beta },\widehat{\alpha })& var(\widehat{\beta })\end{array}\right]$$

where

$$ \begin{aligned} \frac{{\partial^{2} l\left( {\alpha ,\beta } \right)}}{{\partial \alpha^{2} }} & = \frac{ - m}{{\alpha^{2} }} - \mathop \sum \limits_{i = 1}^{m} \left( {\beta (R_{i} + 1} \right) - 1)\frac{{x_{\left( i \right)}\;^{\alpha } \left( {\log \left( {x_{\left( i \right)} } \right)} \right)^{2} }}{{\left( {1 - x_{\left( i \right)}\;^{\alpha } } \right)^{2} }} \\ \frac{{\partial^{2} l\left( {\alpha ,\beta } \right)}}{{\partial \beta^{2} }} & = { }\frac{ - m}{{\beta^{2} }} \\ \end{aligned} $$

and

$$\frac{{\partial }^{2}l\left(\alpha ,\beta \right)}{\partial \alpha \partial \beta }= -\sum_{i=1}^{m}{(R}_{i}+1)\frac{{{x}_{\left(i\right)}}^{\alpha }\mathrm{log}{(x}_{\left(i\right)})}{\left(1-{{x}_{\left(i\right)}}^{\alpha }\right)}$$

Using (6), an asymptotic $100(1-\gamma )\mathrm{\%}$ confidence interval for the parameters $\alpha$ and $\beta$ can be easily obtained as

$$\widehat{\alpha } \pm {Z}_{\frac{\gamma }{2}}\sqrt{var(\widehat{\alpha })}\;\mathrm{ and }\;\widehat{\beta } \pm {Z}_{\frac{\gamma }{2}}\sqrt{var(\widehat{\beta })}$$

respectively. Here $var(\widehat{\alpha })$ and $var(\widehat{\beta })$ are the elements on the main diagonal of the variance–covariance. Where ${Z}_{\frac{\gamma }{2}}$ is the uppe $\frac{\gamma }{2}$ percentile of the standard normal distribution, so MLE $\sim N(0,{I}^{-1}$).

## Bayesian estimation methods

In this section, the Bayesian estimation (BE) of shape parameters $\alpha$ and $\beta$ denoted by $\widetilde{\alpha }$ and $\widetilde{\beta }$ respectively, are obtained under the assumption that $\alpha$ and $\beta$ are independent random variables with prior distributions Gamma(${a}_{1},{b}_{1}$) and Gamma(${a}_{2},{b}_{2}$) respectively with pdfs:

$$ \begin{gathered} \pi_{1} \left( \alpha \right) \propto \alpha^{{a_{1} - 1}} e^{{ - b_{1} \alpha }} \quad \alpha > 0,a_{1} > 0,b_{1} > 0 \hfill \\ \pi_{2} \left( \beta \right) \propto \beta^{{a_{2} - 1}} e^{{ - b_{2} \beta }} \quad \beta > 0,a_{2} > 0,b_{2} > 0 \hfill \\ \end{gathered} $$

where the hyper parameters ${a}_{1},{b}_{1}$, ${a}_{2},\mathrm{and }{b}_{2}$ are chosen to reflect the prior knowledge about $\alpha$ and $\beta$. The joint prior for $\alpha$ and $\beta$ is given by

7 $$\pi \left(\alpha ,\beta \right)={\pi }_{1}\left(\alpha \right){\pi }_{2}\left(\beta \right) \propto {\alpha }^{{a}_{1}-1} {\beta }^{{a}_{2}-1} {e}^{-{b}_{1}\alpha -{b}_{2}\beta }$$

By applying Bayes' theorem and combining the likelihood function from (4) with the joint prior from (7), we can obtain the posterior distribution of the parameters $\alpha$ and $\beta$ denoted as $\pi \left(\alpha ,\beta |x\right)$, which is proportional to the likelihood and prior. This can be expressed as:

$$\pi \left(\alpha ,\beta |x\right)=\frac{\pi \left(\alpha ,\beta \right)L\left(\alpha ,\beta \right)}{{\int }_{0}^{\infty }\underset{0}{\overset{\infty }{\int }}\pi \left(\alpha ,\beta \right)L\left(\alpha ,\beta \right)d\alpha d\beta }$$

when the likelihood function of $\alpha$ and $\beta$ as follows:

$$L\left(\alpha ,\beta \right)= C{(\alpha \beta )}^{m}\prod_{i=1}^{m}{{x}_{(i)}}^{\alpha -1}{\left(1-{{x}_{(i)}}^{\alpha }\right)}^{\beta {(R}_{i}+1)-1}$$

thus, the likelihood function in (4) can be rewritten as follows:

$$ \begin{aligned} L\left( {\alpha ,\beta } \right) & = C\left( {\alpha \beta } \right)^{m} \mathop \prod \limits_{i = 1}^{m} x_{\left( i \right)}\;^{\alpha - 1} \left( {1 - x_{\left( i \right)}\;^{\alpha } } \right)^{{\beta (R_{i} + 1)}} \left( {1 - x_{\left( i \right)}\;^{\alpha } } \right)^{ - 1} \\ & = C\left( {\alpha \beta } \right)^{m} \mathop \prod \limits_{i = 1}^{m} \frac{{x_{\left( i \right)}\;^{\alpha - 1} }}{{\left( {1 - x_{\left( i \right)}\;^{\alpha } } \right)}}\left( {1 - x_{\left( i \right)}\;^{\alpha } } \right)^{{\beta (R_{i} + 1)}} \\ & = C\left( {\alpha \beta } \right)^{m} \mathop \prod \limits_{i = 1}^{m} \frac{{x_{\left( i \right)}\;^{\alpha - 1} }}{{\left( {1 - x_{\left( i \right)}\;^{\alpha } } \right)}}\mathop \prod \limits_{i = 1}^{m} \left( {1 - x_{\left( i \right)}\;^{\alpha } } \right)^{{\beta (R_{i} + 1)}} \\ \end{aligned} $$

Hence, the taken $\mathrm{exp}(\mathrm{log} )$ function will be

$$ \begin{aligned} {\text{exp}}\left( {\log L\left( {\alpha ,\beta } \right)} \right) & = {\text{exp}}\left( {\log \left( {C\left( {\alpha \beta } \right)^{m} \mathop \prod \limits_{i = 1}^{m} \frac{{x_{\left( i \right)}\;^{\alpha - 1} }}{{\left( {1 - x_{\left( i \right)}\;^{\alpha } } \right)}}\mathop \prod \limits_{i = 1}^{m} \left( {1 - x_{\left( i \right)}\;^{\alpha } } \right)^{{\beta (R_{i} + 1)}} } \right)} \right) \\ \therefore L\left( {\alpha ,\beta } \right) & = C\left( {\alpha \beta } \right)^{m} \left[ {\mathop \prod \limits_{i = 1}^{m} \frac{{x_{\left( i \right)}\;^{\alpha - 1} }}{{\left( {1 - x_{\left( i \right)}\;^{\alpha } } \right)}}} \right]\exp \left\{ {{\text{log}}\left( {\mathop \prod \limits_{i = 1}^{m} \left( {1 - x_{\left( i \right)}\;^{\alpha } } \right)^{{\beta (R_{i} + 1)}} } \right)} \right\} \\ & = C\left( {\alpha \beta } \right)^{m} \left[ {\mathop \prod \limits_{i = 1}^{m} \frac{{x_{\left( i \right)}\;^{\alpha - 1} }}{{\left( {1 - x_{\left( i \right)}\;^{\alpha } } \right)}}} \right]\exp \left\{ {\beta \mathop \sum \limits_{i = 1}^{m} (R_{i} + 1)\log \left( {1 - x_{\left( i \right)}\;^{\alpha } } \right)} \right\} \\ \end{aligned} $$

The join posterior density function of $\alpha$ and $\beta$ can be written as:

$$\pi \left(\alpha ,\beta |x\right)={K}^{-1}\left[{\alpha }^{{m+a}_{1}-1} {\beta }^{{m+a}_{2}-1} \left[\prod_{i=1}^{m}\frac{{{x}_{(i)}}^{\alpha -1}}{\left(1-{{x}_{(i)}}^{\alpha }\right)}\right]\mathrm{exp}\left\{-\beta \left({b}_{2}-\sum_{i=1}^{m}{(R}_{i}+1)\mathrm{log}\left(1-{{x}_{(i)}}^{\alpha }\right)\right)-{b}_{1}\alpha \right\}\right]$$

where:

$$K={\int }_{0}^{\infty }{\int }_{0}^{\infty }{\alpha }^{{m+a}_{1}-1} {\beta }^{{m+a}_{2}-1} \left[\prod_{i=1}^{m}\frac{{{x}_{(i)}}^{\alpha -1}}{\left(1-{{x}_{(i)}}^{\alpha }\right)}\right]\mathrm{exp}\left\{-\beta \left({b}_{2}-\sum_{i=1}^{m}{(R}_{i}+1)\mathrm{log}\left(1-{{x}_{(i)}}^{\alpha }\right)\right)-{b}_{1}\alpha \right\} d\alpha d\beta $$

Thus, the posterior density function can be rewritten as

8 $$\pi \left(\alpha ,\beta |x\right)\propto {\alpha }^{{m+a}_{1}-1} {\beta }^{{m+a}_{2}-1}\left[\prod_{i=1}^{m}\frac{{{x}_{(i)}}^{\alpha -1}}{\left(1-{{x}_{(i)}}^{\alpha }\right)}\right]\mathrm{exp}\left\{-\beta \left({b}_{2}-\sum_{i=1}^{m}{(R}_{i}+1)\mathrm{log}\left(1-{{x}_{(i)}}^{\alpha }\right)\right)-{b}_{1}\alpha \right\}$$

The conditional posterior densities of $\alpha$ and $\beta$ are as follows:

$${\pi }_{1}\left(\alpha |\beta ,x\right)=\frac{ \pi \left(\alpha ,\beta |x\right)}{{\pi }_{2}\left(\beta \right)}$$

where

$${\pi }_{2}\left(\beta \right)\propto {\beta }^{m+{a}_{2}-1} {e}^{-{b}_{2}\beta }{\int }_{0}^{\infty }{\alpha }^{m+{a}_{1}-1}\left[\prod_{i=1}^{m}\frac{{{x}_{(i)}}^{\alpha -1}}{\left(1-{{x}_{(i)}}^{\alpha }\right)}\right] \mathrm{exp}\left\{\sum_{i=1}^{m}{(R}_{i}+1)\mathrm{log}\left(1-{{x}_{(i)}}^{\alpha }\right)-{b}_{1}\alpha \right\}d\alpha $$

Hence, the conditional posterior density of $\alpha$ will be

9 $${\pi }_{1}\left(\alpha |\beta ,x\right)\propto {\alpha }^{m+{a}_{1}-1}\left[\prod_{i=1}^{m}\frac{{{x}_{(i)}}^{\alpha -1}}{\left(1-{{x}_{(i)}}^{\alpha }\right)}\right]\mathrm{exp}\left\{\sum_{i=1}^{m}{(R}_{i}+1)\mathrm{log}\left(1-{{x}_{(i)}}^{\alpha }\right)-{b}_{1}\alpha \right\}$$

and

$${\pi }_{2}\left(\beta |\alpha ,x\right)=\frac{ \pi \left(\alpha ,\beta |x\right)}{{\pi }_{1}\left(\alpha \right)}$$

where

$${\pi }_{1}\left(\alpha \right)\propto {\alpha }^{m+{a}_{1}-1} {e}^{-{b}_{1}\alpha }\left[\prod_{i=1}^{m}\frac{{{x}_{(i)}}^{\alpha -1}}{\left(1-{{x}_{(i)}}^{\alpha }\right)}\right]{\int }_{0}^{\infty }{\beta }^{m+{a}_{2}-1} \mathrm{exp}\left\{-\beta \left({b}_{2}-\sum_{i=1}^{m}{(R}_{i}+1)\mathrm{log}\left(1-{{x}_{(i)}}^{\alpha }\right)\right)\right\}d\beta $$

Hence, the conditional posterior density of $\beta$ will be

10 $${\pi }_{2}\left(\beta |\alpha ,x\right)\propto {\beta }^{m+{a}_{2}-1} \mathrm{exp}\left\{-\beta \left({b}_{2}-\sum_{i=1}^{m}{(R}_{i}+1)\mathrm{log}\left(1-{{x}_{(i)}}^{\alpha }\right)\right)\right\}$$

It is clear that ${\pi }_{2}\left(\beta |\alpha ,x\right)$ is the density function of a gamma $(m+{a}_{2}, {b}_{2}-\sum_{i=1}^{m}{(R}_{i}+1)\mathrm{log}\left(1-{{x}_{i}}^{\alpha }\right))$ random variable. BE are obtained based on three different types of loss functions, namely; the squared error (SE) loss function (as a symmetric loss function), linear exponential (LINEX) and general entropy (GE) loss functions (as asymmetric loss functions).

- **SE loss function**

The SE loss function is a symmetric loss function and takes the form

$${g}_{\mathrm{SE}}\left(\widehat{\theta }-\theta \right)\propto {\left(\widehat{\theta }-\theta \right)}^{2},$$

where $\widehat{\theta }$ is the estimate of the parameter $\theta$. Under the SE loss function, the BE ${\widehat{\theta }}_{\mathrm{SE}}$ of $\theta$ is given by

$${\widehat{\theta }}_{\mathrm{SE}}={E}_{\theta }(\theta |x)$$

where ${E}_{\theta }$ stands for posterior expectation. The BE for the parameters $\alpha$ and $\beta$ of the Kum. distribution under SE loss function is the posterior mean, we have

$${\widetilde{\alpha }}_{\mathrm{SE}}={E}_{\alpha }\left(\alpha |x\right)= {\int }_{0}^{\infty }{\int }_{0}^{\infty }\alpha \pi \left(\alpha ,\beta |x\right)d\alpha d\beta $$

and

$${\widetilde{\beta }}_{\mathrm{SE}}={E}_{\beta }\left(\beta |x\right)= {\int }_{0}^{\infty }{\int }_{0}^{\infty }\beta \pi \left(\alpha ,\beta |x\right)d\alpha d\beta $$

- **LINEX loss function**

Zellner^16^ represent the LINEX is an asymmetric loss function defined as

$${g}_{\mathrm{LINEX}}\left(\widehat{\theta }-\theta \right)\propto {e}^{c\left(\widehat{\theta }-\theta \right)}-c\left(\widehat{\theta }-\theta \right)-1,$$

where $\widehat{\theta }$ is the estimate of the parameter $\theta$. Under the LINEX loss function, the BE ${\widehat{\theta }}_{\mathrm{LINEX}}$ of $\theta$ is given by

$${\widehat{\theta }}_{\mathrm{LINEX}}=-\frac{1}{c}\mathrm{log}{E}_{\theta }({e}^{-c\theta }|x)$$

where ${E}_{\theta }$ stands for posterior expectation. The parameter $c$ represents the deviation direction, and the degree of deviation is reflected by its magnitude. When $c < 0$, the underestimation is greater than the overestimation and the opposite is the case when $c > 0$. As $c$ approaches zero, the LINEX loss function can be transformed into the SE loss function. The BE for the parameters $\alpha$ and $\beta$ of the Kum. distribution under LINEX loss function may be defined as

$${\widetilde{\alpha }}_{\mathrm{LINEX}}=-\frac{1}{c}\mathrm{log}{E}_{\alpha }\left({e}^{-c\alpha }|x\right),$$

where

$${E}_{\alpha }\left({e}^{-c\alpha }|x\right)= {\int }_{0}^{\infty }{\int }_{0}^{\infty }{e}^{-c\alpha } \pi \left(\alpha ,\beta |x\right)d\alpha d\beta $$

and

$${\widetilde{\beta }}_{\mathrm{LINEX}}=-\frac{1}{c}\mathrm{log}{E}_{\beta }\left({e}^{-c\beta }|x\right),$$

where

$${E}_{\beta }\left({e}^{-c\beta }|x\right)= {\int }_{0}^{\infty }{\int }_{0}^{\infty }{e}^{-c\beta } \pi \left(\alpha ,\beta |x\right)d\alpha d\beta $$

- **GE loss function**

This asymmetric loss function is another valuable tool for detecting overestimation or underestimation, and it's an extension of the entropy loss function. Calabria and Pulcini^17^ proposed the general entropy loss function with parameter $q$ is given by

$${g}_{\mathrm{GE}}\left(\widehat{\theta }-\theta \right)\propto {\left(\frac{\widehat{\theta }}{\theta }\right)}^{q}-q\mathrm{log}\left(\frac{\widehat{\theta }}{\theta }\right)-1,$$

where $\widehat{\theta }$ is the estimate of the parameter $\theta$. Under the GE loss function, the BE ${\widehat{\theta }}_{\mathrm{GE}}$ of $\theta$ is given by

$${\widehat{\theta }}_{\mathrm{GE}}={\left[{E}_{\theta }({\theta }^{-q}|x)\right]}^{\frac{-1}{q}},$$

where ${E}_{\theta }$ stands for posterior expectation. The proper choice for $q$ is a challenging task for an analyst, because it reflects the asymmetry of the loss function. The BE for the parameters $\alpha$ and $\beta$ of the Kum. distribution under GE loss function may be defined as

$${\widehat{\alpha }}_{\mathrm{GE}}={\left[{E}_{\alpha }({\alpha }^{-q}|x)\right]}^{\frac{-1}{q}},$$

where

$${E}_{\alpha }\left({\alpha }^{-q}|x\right)= {\int }_{0}^{\infty }{\int }_{0}^{\infty }{\alpha }^{-q} \pi \left(\alpha ,\beta |x\right)d\alpha d\beta $$

and

$${\widetilde{\beta }}_{\mathrm{GE}}={\left[{E}_{\beta }({\beta }^{-q}|x)\right]}^{\frac{-1}{q}},$$

where

$${E}_{\beta }\left({\beta }^{-q}|x\right)= {\int }_{0}^{\infty }{\int }_{0}^{\infty }{\beta }^{-q} \pi \left(\alpha ,\beta |x\right)d\alpha d\beta $$

After examining the BE equations presented above, it becomes apparent that they are all the ratios of two integrals and obtaining explicit expressions for these integrals is difficult. Consequently, it is necessary to use appropriate techniques to approximate these integrals. Thus, we provide the approximate BE of $\alpha$ and $\beta$ such as:

- Lindley’s approximation
- Markov chain Monte Carlo (MCMC)

### Lindley’s approximation

Lindley suggested an estimate for calculating the ratio of integrals, as given in (8), using three distinct loss functions, which is based on the prior distributions of $\alpha$ and $\beta$. Consider the ratio of integral $I(X)$, where

$$I\left(X\right)= \frac{{\int }_{0}^{\infty }{\int }_{0}^{\infty }g(\alpha ,\beta ){e}^{l\left(\alpha ,\beta \right)+\rho (\alpha ,\beta )}d\alpha d\beta }{{\int }_{0}^{\infty }{\int }_{0}^{\infty }{e}^{l\left(\alpha ,\beta \right)+\rho (\alpha ,\beta )}d\alpha d\beta }$$

where $g(\alpha ,\beta )$ is function of $\alpha$ and $\beta$ only and $l\left(\alpha ,\beta \right)$ is the log-likelihood and $\rho \left(\alpha ,\beta \right)=\mathrm{log}\pi \left(\alpha ,\beta \right)$. Using the approach developed in Lindley^18^, the ratio of integral $I(X)$ can be written as

$$ \begin{aligned} & I\left( X \right) = \hat{g}\left( {\alpha ,\beta } \right) \\ & \quad + \frac{1}{2}\left[ {\left( {\hat{g}_{\alpha \alpha } + 2\hat{g}_{\alpha } \hat{\rho }_{\alpha } } \right)\hat{\sigma }_{\alpha \alpha } + \left( {\hat{g}_{\beta \alpha } + 2\hat{g}_{\beta } \hat{\rho }_{\alpha } } \right)\hat{\sigma }_{\beta \alpha } } \right. \\ & \quad \left. { + \left( {\hat{g}_{\alpha \beta } + 2\hat{g}_{\alpha } \hat{\rho }_{\beta } } \right)\hat{\sigma }_{\alpha \beta } + \left( {\hat{g}_{\beta \beta } + 2\hat{g}_{\beta } \hat{\rho }_{\beta } } \right)\hat{\sigma }_{\beta \beta } } \right] \\ & \quad + \frac{1}{2}\left[ {\left( {\hat{g}_{\alpha } \hat{\sigma }_{\alpha \alpha } + \hat{g}_{\beta } \hat{\sigma }_{\alpha \beta } } \right)\left( {\hat{l}_{\alpha \alpha \alpha } \hat{\sigma }_{\alpha \alpha } + \hat{l}_{\alpha \beta \alpha } \hat{\sigma }_{\alpha \beta } + \hat{l}_{\beta \alpha \alpha } \hat{\sigma }_{\beta \alpha } + \hat{l}_{\beta \beta \alpha } \hat{\sigma }_{\beta \beta } } \right)} \right. \\ & \quad \left. { + \left( {\hat{g}_{\alpha } \hat{\sigma }_{\beta \alpha } + \hat{g}_{\beta } \hat{\sigma }_{\beta \beta } } \right)\left( {\hat{l}_{\beta \alpha \alpha } \hat{\sigma }_{\alpha \alpha } + \hat{l}_{\alpha \beta \beta } \hat{\sigma }_{\alpha \beta } + \hat{l}_{\beta \alpha \beta } \hat{\sigma }_{\beta \alpha } + \hat{l}_{\beta \beta \beta } \hat{\sigma }_{\beta \beta } } \right)} \right] \\ \end{aligned} $$

where

$${\widehat{g}}_{i}={\left.\frac{\partial g(\alpha ,\beta )}{\partial i}\right|}_{\alpha =\widehat{\alpha },\beta =\widehat{\beta }}; {\widehat{g}}_{ij}={\left.\frac{{\partial }^{2}g(\alpha ,\beta )}{\partial i\partial j}\right|}_{\alpha =\widehat{\alpha },\beta =\widehat{\beta }}$$

$${\widehat{l}}_{ij}={\left.\frac{{\partial }^{2}l(\alpha ,\beta )}{\partial i\partial j}\right|}_{\alpha =\widehat{\alpha },\beta =\widehat{\beta }}; {\widehat{l}}_{ijk}={\left.\frac{{\partial }^{3}l(\alpha ,\beta )}{\partial i\partial j\partial k}\right|}_{\alpha =\widehat{\alpha },\beta =\widehat{\beta }}$$

$${\widehat{\rho }}_{i}={\left.\frac{\partial \rho (\alpha ,\beta )}{\partial i}\right|}_{\alpha =\widehat{\alpha },\beta =\widehat{\beta }};\widehat{\sigma }=-\frac{1}{\widehat{l}}$$

$\widehat{\alpha }$ and $\widehat{\beta }$ are the MLE of $\alpha$ and $\beta$ and then, partial derivatives of log-likelihood function of ${L}_{ijk}$ (5), one can be obtained as follows:

$${l}_{\alpha }=\frac{\partial l\left(\alpha ,\beta \right)}{\partial \alpha }=\frac{m}{\alpha }+\sum_{i=1}^{m}\mathrm{log}{(x}_{(i)})-\sum_{i=1}^{m}(\beta {(R}_{i}+1)-1)\frac{{{x}_{\left(i\right)}}^{\alpha }\mathrm{log}{(x}_{\left(i\right)})}{\left(1-{{x}_{\left(i\right)}}^{\alpha }\right)}$$

$${l}_{\alpha \alpha }=\frac{{\partial }^{2}l\left(\alpha ,\beta \right)}{\partial {\alpha }^{2}} = \frac{-m}{{\alpha }^{2}}-\sum_{i=1}^{m}(\beta {(R}_{i}+1)-1)\frac{{{x}_{\left(i\right)}}^{\alpha }{\left(\mathrm{log}\left({x}_{\left(i\right)}\right)\right)}^{2}}{{\left(1-{{x}_{\left(i\right)}}^{\alpha }\right)}^{2}}$$

$${l}_{\alpha \alpha \alpha }=\frac{{\partial }^{3}l\left(\alpha ,\beta \right)}{\partial {\alpha }^{3}}= \frac{2\mathrm{m}}{{\alpha }^{3}}-\sum_{i=1}^{m}(\beta {(R}_{i}+1)-1)$$

$$\frac{{{x}_{(i)}}^{\alpha }{\left(1-{{x}_{(i)}}^{\alpha }\right)}^{2}{\left(\mathrm{log}{(x}_{(i)})\right)}^{3}+2{\left({{x}_{(i)}}^{\alpha }\right)}^{2}\left(1-{{x}_{(i)}}^{\alpha }\right){\left(\mathrm{log}{(x}_{(i)})\right)}^{3}}{{\left(1-{{x}_{(i)}}^{\alpha }\right)}^{4}}$$

$${l}_{\beta }=\frac{\partial l\left(\alpha ,\beta \right)}{\partial \beta }= \frac{m}{\beta }+\sum_{i=1}^{m}{(R}_{i}+1)\mathrm{log}(1-{{x}_{(i)}}^{\alpha })$$

$${l}_{\beta \beta }=\frac{{\partial }^{2}l\left(\alpha ,\beta \right)}{\partial {\beta }^{2}}= \frac{-m}{{\beta }^{2}}$$

$${l}_{\beta \beta \beta }=\frac{{\partial }^{2}l\left(\alpha ,\beta \right)}{\partial {\beta }^{3}}= \frac{2m}{{\beta }^{3}}$$

$${l}_{\alpha \beta }=\frac{{\partial }^{2}l\left(\alpha ,\beta \right)}{\partial \alpha \partial \beta }=-\sum_{i=1}^{m}{(R}_{i}+1)\frac{{{x}_{(i)}}^{\alpha }\mathrm{log}{(x}_{(i)})}{\left(1-{{x}_{(i)}}^{\alpha }\right)}$$

$${l}_{\alpha \beta \alpha }=\frac{{\partial }^{3}l\left(\alpha ,\beta \right)}{\partial \alpha \partial \beta \partial \alpha }= -\sum_{i=1}^{m}{(R}_{i}+1)\frac{{{x}_{\left(i\right)}}^{\alpha }{\left(\mathrm{log}\left({x}_{\left(i\right)}\right)\right)}^{2}}{{\left(1-{{x}_{\left(i\right)}}^{\alpha }\right)}^{2}}$$

$${l}_{\alpha \beta \beta }=\frac{{\partial }^{3}l\left(\alpha ,\beta \right)}{\partial \alpha \partial \beta \partial \beta }=0$$

The partial derivatives of log-prior function as follows:

$$\rho \left(\alpha ,\beta \right)=\mathrm{log}\pi \left(\alpha ,\beta \right) = C +\left({a}_{1}-1\right)\mathrm{log}\alpha +\left({a}_{2}-1\right)\mathrm{log}\beta - {b}_{1}\alpha -{b}_{2}\beta $$

thus,

$${\rho }_{\alpha }=\frac{{a}_{1}-1}{\alpha }-{b}_{1}, {\rho }_{\beta }=\frac{{a}_{2}-1}{\beta }-{b}_{2}$$

#### Lindley’s approximation SE loss function

When $g\left(\alpha ,\beta \right)= \alpha$, we observe that

$${g}_{\alpha }=1, {g}_{\beta }={g}_{\alpha \alpha }={g}_{\alpha \beta }={g}_{\beta \alpha }={g}_{\beta \beta }=0\; \& \;{l}_{\alpha \beta \beta }=0$$

hence, the BE of $\alpha$ can be obtained as

$${\widetilde{\alpha }}_{SE}= \widehat{\alpha }+{\widehat{\rho }}_{\alpha }{\widehat{\sigma }}_{\alpha \alpha }+{\widehat{\rho }}_{\beta }{\widehat{\sigma }}_{\alpha \beta }+\frac{1}{2}\left[{\widehat{\sigma }}_{\alpha \alpha }\left({\widehat{l}}_{\alpha \alpha \alpha }{\widehat{\sigma }}_{\alpha \alpha }+2{\widehat{l}}_{\alpha \beta \alpha }{\widehat{\sigma }}_{\alpha \beta }\right)+{\widehat{\sigma }}_{\beta \alpha }\left({\widehat{l}}_{\beta \alpha \alpha }{\widehat{\sigma }}_{\alpha \alpha }+{\widehat{l}}_{\beta \beta \beta }{\widehat{\sigma }}_{\beta \beta }\right)\right]$$

When $g\left(\alpha ,\beta \right)= \beta$, we can derive that

$${g}_{\beta }=1, {g}_{\alpha }={g}_{\alpha \alpha }={g}_{\alpha \beta }={g}_{\beta \alpha }={g}_{\beta \beta }=0 \;\&\; {l}_{\alpha \beta \beta }=0$$

hence, the BE of $\beta$ can be obtained as

$${\widetilde{\beta }}_{SE}= \widehat{\beta }+{\widehat{\rho }}_{\alpha }{\widehat{\sigma }}_{\beta \alpha }+{\widehat{\rho }}_{\beta }{\widehat{\sigma }}_{\beta \beta }+\frac{1}{2}\left[{\widehat{\sigma }}_{\alpha \beta }\left({\widehat{l}}_{\alpha \alpha \alpha }{\widehat{\sigma }}_{\alpha \alpha }+2{\widehat{l}}_{\beta \alpha \alpha }{\widehat{\sigma }}_{\beta \alpha }\right)+{\widehat{\sigma }}_{\beta \beta }\left({\widehat{l}}_{\beta \alpha \alpha }{\widehat{\sigma }}_{\alpha \alpha }+{\widehat{l}}_{\beta \beta \beta }{\widehat{\sigma }}_{\beta \beta }\right)\right]$$

#### Lindley’s approximation LINEX loss function

When $g\left(\alpha ,\beta \right)= {e}^{-c\alpha }$, we observe that

$${g}_{\alpha }=-c{e}^{-c\alpha }, {g}_{\alpha \alpha }={c}^{2}{e}^{-c\alpha }, {g}_{\beta }={g}_{\alpha \beta }={g}_{\beta \alpha }={g}_{\beta \beta }=0 \& {l}_{\alpha \beta \beta }=0$$

hence, the BE of $\alpha$ can be obtained as

$${\widetilde{\alpha }}_{\mathrm{LINEX}}=-\frac{1}{c}\mathrm{log}\left\{{e}^{-c\widehat{\alpha }}+\frac{1}{2}{\widehat{g}}_{\alpha \alpha }{\widehat{\sigma }}_{\alpha \alpha }+{\widehat{g}}_{\alpha }{\widehat{\rho }}_{\alpha }{\widehat{\sigma }}_{\alpha \alpha }+{\widehat{g}}_{\alpha }{\widehat{\rho }}_{\beta }{\widehat{\sigma }}_{\alpha \beta }+\frac{1}{2}\left[{\widehat{g}}_{\alpha }{\widehat{\sigma }}_{\alpha \alpha }\left({\widehat{l}}_{\alpha \alpha \alpha }{\widehat{\sigma }}_{\alpha \alpha }+2{\widehat{l}}_{\alpha \beta \alpha }{\widehat{\sigma }}_{\alpha \beta }\right)+{\widehat{g}}_{\alpha }{\widehat{\sigma }}_{\beta \alpha }\left({\widehat{l}}_{\beta \alpha \alpha }{\widehat{\sigma }}_{\alpha \alpha }+{\widehat{l}}_{\beta \beta \beta }{\widehat{\sigma }}_{\beta \beta }\right)\right]\right\}$$

When $g\left(\alpha ,\beta \right)= {e}^{-c\beta }$, we observe that

$${g}_{\beta }=-c{e}^{-c\beta }, {g}_{\beta \beta }={c}^{2}{e}^{-c\beta }, {g}_{\alpha }={g}_{\alpha \beta }={g}_{\beta \alpha }={g}_{\alpha \alpha }=0 \& {l}_{\alpha \beta \beta }=0$$

hence, the BE of $\beta$ can be obtained as

$${\widetilde{\beta }}_{\mathrm{LINEX}}=-\frac{1}{c}\mathrm{log}\left\{{e}^{-c\widehat{\beta }}+{\widehat{g}}_{\beta }{\widehat{\rho }}_{\alpha }{\widehat{\sigma }}_{\beta \alpha }+\frac{1}{2}{\widehat{g}}_{\beta \beta }{\widehat{\sigma }}_{\beta \beta }+{\widehat{g}}_{\beta }{\widehat{\rho }}_{\beta }{\widehat{\sigma }}_{\beta \beta }+\frac{1}{2}\left[{\widehat{g}}_{\beta }{\widehat{\sigma }}_{\alpha \beta }\left({\widehat{l}}_{\alpha \alpha \alpha }{\widehat{\sigma }}_{\alpha \alpha }+2{\widehat{l}}_{\beta \alpha \alpha }{\widehat{\sigma }}_{\beta \alpha }\right)+{\widehat{g}}_{\beta }{\widehat{\sigma }}_{\beta \beta }\left({\widehat{l}}_{\beta \alpha \alpha }{\widehat{\sigma }}_{\alpha \alpha }+{\widehat{l}}_{\beta \beta \beta }{\widehat{\sigma }}_{\beta \beta }\right)\right]\right\}$$

#### Lindley’s approximation GE loss function

When $g\left(\alpha ,\beta \right)= {\alpha }^{-q}$, we observe that

$${g}_{\alpha }=-q{\alpha }^{-q-1}, {g}_{\alpha \alpha }=q\left(q+1\right){\alpha }^{-q-2},$$

$${g}_{\beta }={g}_{\alpha \beta }={g}_{\beta \alpha }={g}_{\beta \beta }=0 \& {l}_{\alpha \beta \beta }=0$$

hence, the BE of $\alpha$ can be obtained as

$${\widetilde{\alpha }}_{\mathrm{GE}}={\left\{{\widehat{\alpha }}^{-q}+\frac{1}{2}{\widehat{g}}_{\alpha \alpha }{\widehat{\sigma }}_{\alpha \alpha }+{\widehat{g}}_{\alpha }{\widehat{\rho }}_{\alpha }{\widehat{\sigma }}_{\alpha \alpha }+{\widehat{g}}_{\alpha }{\widehat{\rho }}_{\beta }{\widehat{\sigma }}_{\alpha \beta }+\frac{1}{2}\left[{\widehat{g}}_{\alpha }{\widehat{\sigma }}_{\alpha \alpha }\left({\widehat{l}}_{\alpha \alpha \alpha }{\widehat{\sigma }}_{\alpha \alpha }+2{\widehat{l}}_{\alpha \beta \alpha }{\widehat{\sigma }}_{\alpha \beta }\right)+{\widehat{g}}_{\alpha }{\widehat{\sigma }}_{\beta \alpha }\left({\widehat{l}}_{\beta \alpha \alpha }{\widehat{\sigma }}_{\alpha \alpha }+{\widehat{l}}_{\beta \beta \beta }{\widehat{\sigma }}_{\beta \beta }\right)\right]\right\}}^{\frac{-1}{q}}$$

When $g\left(\alpha ,\beta \right)= {\beta }^{-q}$, we observe that

$${g}_{\beta }=-q{\beta }^{-q-1}, {g}_{\beta \beta }=q\left(q+1\right){\beta }^{-q-2},$$

$${g}_{\alpha }={g}_{\alpha \beta }={g}_{\beta \alpha }={g}_{\alpha \alpha }=0 \& {l}_{\alpha \beta \beta }=0$$

hence, the BE of $\beta$ can be obtained as

$${\widetilde{\beta }}_{GE}={\left\{{\widehat{\beta }}^{-q}+{\widehat{g}}_{\beta }{\widehat{\rho }}_{\alpha }{\widehat{\sigma }}_{\beta \alpha }+\frac{1}{2}{\widehat{g}}_{\beta \beta }{\widehat{\sigma }}_{\beta \beta }+{\widehat{g}}_{\beta }{\widehat{\rho }}_{\beta }{\widehat{\sigma }}_{\beta \beta }+\frac{1}{2}\left[{\widehat{g}}_{\beta }{\widehat{\sigma }}_{\alpha \beta }\left({\widehat{l}}_{\alpha \alpha \alpha }{\widehat{\sigma }}_{\alpha \alpha }+2{\widehat{l}}_{\beta \alpha \alpha }{\widehat{\sigma }}_{\beta \alpha }\right)+{\widehat{g}}_{\beta }{\widehat{\sigma }}_{\beta \beta }\left({\widehat{l}}_{\beta \alpha \alpha }{\widehat{\sigma }}_{\alpha \alpha }+{\widehat{l}}_{\beta \beta \beta }{\widehat{\sigma }}_{\beta \beta }\right)\right]\right\}}^{\frac{-1}{q}}$$

It is hard to obtain the third derivatives of the log-likelihood function, so the Metropolis–Hastings algorithm (MH) is used for computing the desired BE. There are two advantages of considering the MH algorithm over Lindley’s method. Firstly, there is no need to calculate up to the third derivatives of the log-likelihood function. Secondly, the samples obtained through the MH algorithm can be used to obtain highest posterior density (HPD) intervals for the distribution's unknown parameters, which is not possible with Lindley's method, for more details see Dey et al.^19^.

### Markov chain Monte-Carlo

In this case, we apply the MCMC technique to produce samples from the posterior distributions. From these samples, we calculate the BE of the unknown parameters and construct the corresponding credible intervals. he conditional posterior densities of $\beta$ in (10) are gamma densities with a shape parameter of $(m+{a}_{2})$ and a scale parameter of $\left({b}_{2}-\sum_{i=1}^{m}{(R}_{i}+1)\mathrm{log}\left(1-{{x}_{(i)}}^{\alpha }\right)\right)$. Thus, samples of $\beta$ can be easily generated using any gamma generating routine. The posterior of $\alpha$ given in (9) does not present a standard form, but the plot of it shows that it is similar to a normal distribution with mean $\alpha$ and standard deviation ${S}_{\alpha }$, here ${S}_{\alpha }$ represents the variance–covariance matrix. Therefore, to generate random numbers from this distribution, we use the Metropolis–Hastings algorithm with the normal proposal distribution to generate samples from it see Tierney^20^ and El-Sagheer^11^. Therefore, the MCMC is given as:

1. Start initial value of $\theta$ as $\theta ={\theta }^{\left(0\right)}$, where $\theta =(\alpha ,\beta )$.
2. Set $$i=1$$.
3. Generate ${\beta }^{(i)}$ from Gamma $\left(m+{a}_{2}, {b}_{2}-\sum_{i=1}^{m}{(R}_{i}+1)\mathrm{log}\left(1-{{x}_{(i)}}^{\alpha }\right)\right)$.
4. Using the following Metropolis–Hastings, generate ${\alpha }^{(i)}$ from ${\pi }_{1}\left(\alpha |\beta ,x\right)$ with the normal proposal distribution $N\left(\widehat{\alpha }, {S}_{\alpha }\right)$.
  4.1 Generate a proposal ${\alpha }^{*}$ from $N\left(\widehat{\alpha }, {S}_{\alpha }\right)$, where ${S}_{\alpha }$ is Standard deviation of $\alpha$.
  4.2 Calculate the acceptance probability $\rho$ where $\rho =\mathrm{min}\left(1, \frac{{\pi }_{1}\left({\alpha }^{*}|{\beta }^{(i)},x\right)}{{\pi }_{1}\left({\alpha }^{(i-1)}|{\beta }^{(i)},x\right)}\right)$.
  4.3 Generate $u$ from a uniform (0,1) distribution.
  4.4 If $u< \rho$, accept the proposal and set ${\theta }^{(i)}={\theta }^{*}$, else set ${\theta }^{(i)}={\theta }^{(i-1)}$.
5. Set $$i=i+1$$.
6. Repeat steps 3–6 $N$ times.

After drawing a random sample of size $M$ from the posterior density, it's possible to discard some initial samples (burn-in) and then use the remaining samples to calculate BE of $\theta =(\alpha , \beta )$. By doing this, it's possible to derive approximate Bayes point estimates of $\alpha$ and $\beta$ under the SE, LINEX, and GE loss functions as follows.

$${\widetilde{g}}_{SE}\left(\alpha ,\beta \right)=\frac{1}{M-{l}_{B}}\sum_{i={l}_{B}}^{M}{\theta }^{\left(i\right)}$$

$${\widetilde{g}}_{\mathrm{LINEX}}\left(\alpha ,\beta \right)=-\frac{1}{c}\mathrm{log}\left(\frac{1}{M-{l}_{B}}\sum_{i={l}_{B}}^{M}{e}^{-c{\theta }^{\left(i\right)}}\right)$$

and

$${\widetilde{g}}_{\mathrm{GE}}\left(\alpha ,\beta \right)={\left(\frac{1}{M-{l}_{B}}\sum_{i={l}_{B}}^{M}{\left({\theta }^{(i)}\right)}^{-q}\right)}^{\frac{-1}{q}}$$

where ${l}_{B}$ represents the number of burn-in samples. For computation of the $100(1-\gamma )\mathrm{\%}$ HPD interval of $\theta$, order ${\theta }^{(1)}, {\theta }^{(2)}, \dots , {\theta }^{(M)}$ as ${\theta }^{(1)}< {\theta }^{(2)}< \dots <{\theta }^{(M)}$ . Then construct all the $100(1-\gamma )\mathrm{\%}$ confidence intervals of $\theta$, as:

$$\left[{\theta }^{(1)}, {\theta }^{(N\left[1-\gamma \right]+1)}\right], \left[{\theta }^{(2)}, {\theta }^{(N\left[1-\gamma \right]+2)}\right], \dots , \left[{\theta }^{(N)}, {\theta }^{\left[N\gamma \right]}\right]$$

Finally, the HPD confidence interval of $\alpha$ and $\beta$ is that interval which has the shortest length.

### Elicitation of hyper-parameters

This section discusses the elicitation of hyper-parameter values when informative priors are considered. It is to be noted that the hyper-parameter values can be chosen depending on informative priors. Suppose that we have $k$ samples available from the $K\left(\alpha ,\beta \right)$ distribution, and that the associated maximum likelihood estimates of $\left(\alpha ,\beta \right)$ be $\left({\widehat{\alpha }}^{j}, {\widehat{\beta }}^{j}\right), j=\mathrm{1,2},\dots ,k$. The hyper-parameter values can now be obtained by equating the mean and variance of $\left({\widehat{\alpha }}^{j}, {\widehat{\beta }}^{j}\right), j=\mathrm{1,2},\dots ,k$ with the mean and variance of examined priors. In the present work, we have considered the gamma prior of $\alpha$ and $\beta$ respectively are ${\pi }_{1}\left(\alpha \right)\propto {\alpha }^{{a}_{1}-1} {e}^{-{b}_{1}\alpha }$ and ${\pi }_{2}(\beta )\propto {\beta }^{{a}_{2}-1} {e}^{-{b}_{2}\beta }$ for which $\mathrm{Mean}\left(\alpha \right)=\frac{{a}_{1}}{{b}_{1}}$ , $\mathrm{Mean}\left(\beta \right)=\frac{{a}_{2}}{{b}_{2}}$ and $\mathrm{Variance}\left(\alpha \right)=\frac{{a}_{1}}{{{b}_{1}}^{2}}$ , $\mathrm{Variance}\left(\beta \right)=\frac{{a}_{2}}{{{b}_{2}}^{2}}$. Therefore, on equating mean and variance of $\left({\widehat{\alpha }}^{j}, {\widehat{\beta }}^{j}\right), j=\mathrm{1,2},\dots ,k$ with the mean and variance of gamma priors, we get

11 $$\frac{1}{k}\sum_{j=1}^{k}{\widehat{\alpha }}^{j}=\frac{{a}_{1}}{{b}_{1}}, \;\mathrm{and}\; \frac{1}{k-1}\sum_{j=1}^{k}{\left({\widehat{\alpha }}^{j}-\frac{1}{k}\sum_{j=1}^{k}{\widehat{\alpha }}^{j}\right)}^{2}=\frac{{a}_{1}}{{{b}_{1}}^{2}}$$

12 $$\frac{1}{k}\sum_{j=1}^{k}{\widehat{\beta }}^{j}=\frac{{a}_{2}}{{b}_{2}}, \;\mathrm{and}\;\frac{1}{k-1}\sum_{j=1}^{k}{\left({\widehat{\beta }}^{j}-\frac{1}{k}\sum_{j=1}^{k}{\widehat{\beta }}^{j}\right)}^{2}=\frac{{a}_{2}}{{{b}_{2}}^{2}}$$

We can find ${\widehat{a}}_{1}$ and ${\widehat{b}}_{1}$, estimators for $a$ and $b$, by solving Eqs. (11) as follows:

$$\frac{1}{k}\sum_{j=1}^{k}{\widehat{\alpha }}^{j}=\frac{{a}_{1}}{{b}_{1}} \to {a}_{1}= {b}_{1}\frac{1}{k}\sum_{j=1}^{k}{\widehat{\alpha }}^{j}$$

and

$$\frac{1}{k-1}\sum_{j=1}^{k}{\left({\widehat{\alpha }}^{j}-\frac{1}{k}\sum_{j=1}^{k}{\widehat{\alpha }}^{j}\right)}^{2}=\frac{{a}_{1}}{{{b}_{1}}^{2}} \to {a}_{1}= {{b}_{1}}^{2}\frac{1}{k-1}\sum_{j=1}^{k}{\left({\widehat{\alpha }}^{j}-\frac{1}{k}\sum_{j=1}^{k}{\widehat{\alpha }}^{j}\right)}^{2}$$

Solving the above equations yields the estimators for the hyper-parameters

$${b}_{1}=\frac{\frac{1}{k}\sum_{j=1}^{k}{\widehat{\alpha }}^{j}}{\frac{1}{k-1}\sum_{j=1}^{k}{\left({\widehat{\alpha }}^{j}-\frac{1}{k}\sum_{j=1}^{k}{\widehat{\alpha }}^{j}\right)}^{2}}, \;\mathrm{and}\;{a}_{1}=\frac{{\left(\frac{1}{k}\sum_{j=1}^{k}{\widehat{\alpha }}^{j}\right)}^{2}}{\frac{1}{k-1}\sum_{j=1}^{k}{\left({\widehat{\alpha }}^{j}-\frac{1}{k}\sum_{j=1}^{k}{\widehat{\alpha }}^{j}\right)}^{2}}$$

for the prior distribution for $\alpha$. Similarly, estimators for the hyper-parameters for the prior distribution for $\beta$ can be found as

$${a}_{2}=\frac{{\left(\frac{1}{k}\sum_{j=1}^{k}{\widehat{\beta }}^{j}\right)}^{2}}{\frac{1}{k-1}\sum_{j=1}^{k}{\left({\widehat{\beta }}^{j}-\frac{1}{k}\sum_{j=1}^{k}{\widehat{\beta }}^{j}\right)}^{2}}, \;\mathrm{and}\;{b}_{2}=\frac{\frac{1}{k}\sum_{j=1}^{k}{\widehat{\beta }}^{j}}{\frac{1}{k-1}\sum_{j=1}^{k}{\left({\widehat{\beta }}^{j}-\frac{1}{k}\sum_{j=1}^{k}{\widehat{\beta }}^{j}\right)}^{2}}$$

One may also refer to the work of Dey et al.^19,21^, and Singh and Tripathi^22^ in this regard.

## Simulation study and real data analysis

The objective of this section is to evaluate the effectiveness of the different estimation methods that were discussed in the preceding sections. A real dataset is used to illustrative purposes, and a simulation study is conducted to observe how the proposed methods perform under a PCS-II.

### Simulation study

In this subsection, we conduct a simulation study to compare the performance of different estimates and confidence intervals for the Non-BE and BE methods of unknown parameters of the Kum. distribution under a PCS-II. We will compute the Average, Mean Square Error (MSE), Confidence intervals (CI), average interval length (AIL), and the coverage probability (CP) to compare the performance of the different methods using a number of replications 1000. The performance of Non-BE and BE are compared based on the following assumptions:

1. values of $$\left(\alpha , \beta \right)=\left(0.5, 0.5\right), \left(0.5, 1\right), \left(1, 1\right), \;\mathrm{and}\;(1, 2)$$.

2. Sample sizes of *n* = 40 and *n* = 80.

3. In this simulation, the algorithm proposed by Balakrishnan and Sandhu^23^ can be used to generate a progressively Type-II censored sample, removed items ${R}_{i}$ are assumed at different sample sizes $n$ and the number of stages $m$ as shown in Table 1.

**Table 1:** Numerous patterns for removing items from life test at different number of stages.

$n$	$m$	Censoring schemes			
		${S}_{1}$	${S}_{2}$	${S}_{3}$	${S}_{4}$
40	20	$(20, {0}^{*19})$	$(10,{0}^{*18}, 10)$	$({0}^{*9}, \mathrm{10,10},{0}^{*9})$	$( {0}^{*19}, 20)$
	30	$(10, {0}^{*29})$	$(5,{0}^{*28}, 5)$	$({0}^{*14}, \mathrm{5,5},{0}^{*14})$	$( {0}^{*29}, 10)$
80	40	$(40, {0}^{*39})$	$(20,{0}^{*38}, 20)$	$({0}^{*19}, \mathrm{20,20},{0}^{*19})$	$( {0}^{*39}, 40)$
	60	$(20, {0}^{*59})$	$(10,{0}^{*58}, 10)$	$({0}^{*29}, \mathrm{10,10},{0}^{*29})$	$( {0}^{*59}, 20)$

Here, $({2}^{*3},0)$, for example, means that the censoring scheme employed is (2,2,2,0).

Based on the generated data, we compute the MLEs, MPSs, and corresponding 95% asymptotic confidence intervals (Asy-CI). On deriving MLEs, be aware that the initial assume values are considered as true parameter values.

The gamma prior distribution is used to compute BE parameter using both symmetric and asymmetric loss functions. These estimates are obtained through Lindley's approximation and the MCMC method. To determine the values of the hyper-parameters, 500 complete samples of size 60 are constructed from the Kum. distribution with various values of $\alpha$ and $\beta$ using historical data. The obtained informative prior values are then used to evaluate the desired estimates. The MLEs are employed as initial values using the MH algorithm, along with the corresponding variance–covariance matrix ${S}_{\alpha }$ of $\widehat{\alpha }$. In the end, the posterior density removed 2000 burn-in samples of the total of 10,000 generated samples and produced BE for three different loss functions: SE, LINEX at $c = -0.5, 1.5$, and GE loss functions at $q = 0.1, 2$. Additionally, HPD interval estimates were calculated using the approach developed by Chen and Shao^24^.

In Tables 2, 3, 4, 5, we display the Non-BE obtained by using MLE and MPS at different values of $n$ and $m$, respectively. Further, the first column represents the average estimates (Avg.) and the second column represents the mean square errors (MSEs). For confidence intervals, we have asymptotic confidence interval (Asy CI), average interval length (AIL), and coverage probability (CP) using the MLE.

**Table 2:** Avg., MSE (in practices), Asy-CI, AILs and CPs (in %) for Non-BEs of the Kum. distribution for different PCS-II when $\alpha =0.5$ , $\beta =0.5$ at different values of $n$ and $m$.

$(n,m)$	Sch	Parm	Non-Bayesian		Asy CI	
			MLE	MPS	(Lower, Upper)	AIL/CP
(40, 20)	${S}_{1}$	$\alpha$ $\beta$	0.5478 (0.0308) 0.5553 (0.0339)	0.6925 (0.0919) 0.6665 (0.0812)	(0.2276, 0.8681) (0.2659, 0.8448)	0.6403/94.7 0.5788/93.5
	${S}_{2}$	$\alpha$ $\beta$	0.5592 (0.0341) 0.5764 (0.0522)	0.6379 (0.0621) 0.6164 (0.0746)	(0.2352, 0.8831) (0.2361, 0.9168)	0.6478/94.6 0.6807/93.1
	${S}_{3}$	$\alpha$ $\beta$	0.5556 (0.0303) 0.5789 (0.0554)	0.6697 (0.0693) 0.7133 (0.1368)	(0.2541, 0.8573) (0.2360, 0.9219)	0.6032/94.9 0.6858/92.9
	${S}_{4}$	$\alpha$ $\beta$	0.5680 (0.0374) 0.5993 (0.0772)	0.6335 (0.0602) 0.6382 (0.1082)	(0.2378, 0.8982) (0.2014, 0.9973)	0.6604/94.2 0.7959/92.7
(40, 30)	${S}_{1}$	$\alpha$ $\beta$	0.5566 (0.0299) 0.5406 (0.0194)	0.6593 (0.0645) 0.6137 (0.0388)	(0.2614, 0.8518) (0.3104, 0.7708)	0.5903/94.9 0.4604/93.9
	${S}_{2}$	$\alpha$ $\beta$	0.5596 (0.0305) 0.5461 (0.0222)	0.6270 (0.0507) 0.5780 (0.0299)	(0.2660, 0.8532) (0.3036, 0.7886)	0.5871/94.7 0.4850/93.6
	${S}_{3}$	$\alpha$ $\beta$	0.5582 (0.0293) 0.5475 (0.0230)	0.6511 (0.0584) 0.6259 (0.0468)	(0.2732, 0.8432) (0.3016, 0.7933)	0.5700/95.0 0.4916/93.7
	${S}_{4}$	$\alpha$ $\beta$	0.5623 (0.0313) 0.5518 (0.0255)	0.6209 (0.0483) 0.5780 (0.0325)	(0.2692, 0.8555) (0.2962, 0.8075)	0.5863/94.8 0.5113/93.6
(80, 40)	${S}_{1}$	$\alpha$ $\beta$	0.5267 (0.0139) 0.5246 (0.0116)	0.6050 (0.0299) 0.5803 (0.0213)	(0.2984, 0.7550) (0.3338, 0.7153)	0.4565/96.3 0.3815/94.7
	${S}_{2}$	$\alpha$ $\beta$	0.5314 (0.0141) 0.5336 (0.0156)	0.5749 (0.0214) 0.5539 (0.0194)	(0.3074, 0.7553) (0.3167, 0.7505)	0.4478/96.1 0.4337/94.4
	${S}_{3}$	$\alpha$ $\beta$	0.5286 (0.0122) 0.5342 (0.0159)	0.5899 (0.0224) 0.5987 (0.0306)	(0.3224, 0.7349) (0.3157, 0.7528)	0.4125/95.5 0.4371/94.4
	${S}_{4}$	$\alpha$ $\beta$	0.5348 (0.0146) 0.5429 (0.0206)	0.5713 (0.0206) 0.5625 (0.0252)	(0.3113, 0.7584) (0.2984, 0.7874)	0.4470/95.7 0.4890/94.3
(80, 60)	${S}_{1}$	$\alpha$ $\beta$	0.5277 (0.0127) 0.5189 (0.0078)	0.5835 (0.0219) 0.5573 (0.0123)	(0.3247, 0.7308) (0.3639, 0.6739)	0.4061/95.3 0.3099/95.2
	${S}_{2}$	$\alpha$ $\beta$	0.5285 (0.0125) 0.5212 (0.0086)	0.5654 (0.0177) 0.5380 (0.0104)	(0.3287, 0.7282) (0.3595, 0.6830)	0.3995/95.4 0.3235/94.9
	${S}_{3}$	$\alpha$ $\beta$	0.5272 (0.0115) 0.5217 (0.0088)	0.5773 (0.0189) 0.5624 (0.0142)	(0.3344, 0.7201) (0.3579, 0.6856)	0.3857/95.3 0.3276/95.0
	${S}_{4}$	$\alpha$ $\beta$	0.5292 (0.0123) 0.5237 (0.0095)	0.5616 (0.0167) 0.5379 (0.0112)	(0.3316, 0.7268) (0.3548, 0.6926)	0.3952/95.3 0.3378/94.7

Sch.—scheme, Parm.—Parameter.

**Table 3:** Avg., MSE (in practices), Asy-CI, AILs and CPs (in %) for Non-BEs of the Kum. distribution for different PCS-II when $\alpha =0.5$ , $\beta =1$ at different values of $n$ and $m$.

$(n,m)$	Sch	Parm	Non-Bayesian		Asy CI	
			MLE	MPS	(Lower, Upper)	AIL/CP
(40, 20)	${S}_{1}$	$\alpha$ $\beta$	0.5362 (0.0202) 1.1375 (0.1923)	0.6467 (0.0524) 1.4239 (0.5218)	(0.2744, 0.7979) (0.4748, 1.8002)	0.5234/95.2 1.3253/93.5
	${S}_{2}$	$\alpha$ $\beta$	0.5475 (0.0237) 1.2076 (0.3543)	0.6057 (0.0390) 1.3238 (0.5548)	(0.2735, 0.8215) (0.3564, 2.0588)	0.5479/94.9 1.7024/92.7
	${S}_{3}$	$\alpha$ $\beta$	0.5427 (0.0199) 1.2023 (0.3478)	0.6355 (0.0438) 1.5564 (0.9635)	(0.2956, 0.7899) (0.3895, 2.0150)	0.4943/95.0 1.6254/92.8
	${S}_{4}$	$\alpha$ $\beta$	0.5560 (0.0269) 1.2812 (0.5882)	0.6063 (0.0405) 1.4027 (0.9064)	(0.2714, 0.8406) (0.2292, 2.3331)	0.5692/94.5 2.1038/92.4
(40, 30)	${S}_{1}$	$\alpha$ $\beta$	0.5413 (0.0183) 1.1029 (0.1068)	0.6202 (0.0367) 1.2881 (0.2362)	(0.3058, 0.7767) (0.5775, 1.6283)	0.4709/95.3 1.0507/93.4
	${S}_{2}$	$\alpha$ $\beta$	0.5449 (0.0193) 1.1219 (0.1327)	0.5929 (0.0292) 1.2043 (0.1872)	(0.3065, 0.7833) (0.5488, 1.6950)	0.4768/95.2 1.1461/93.2
	${S}_{3}$	$\alpha$ $\beta$	0.5427 (0.0180) 1.1213 (0.1326)	0.6162 (0.0344) 1.3223 (0.2971)	(0.3145, 0.7708) (0.5515, 1.6912)	0.4562/94.9 1.1397/93.3
	${S}_{4}$	$\alpha$ $\beta$	0.5479 (0.0203) 1.1405 (0.1615)	0.5900 (0.0289) 1.2111 (0.2156)	(0.3067, 0.7891) (0.5197, 1.7613)	0.4823/95.0 1.2415/93.6
(80, 40)	${S}_{1}$	$\alpha$ $\beta$	0.5204 (0.0092) 1.0608 (0.0594)	0.5819 (0.0184) 1.1992 (0.1207)	(0.3332, 0.7077) (0.6328, 1.4887)	0.3745/96.3 0.8559/94.7
	${S}_{2}$	$\alpha$ $\beta$	0.5254 (0.0100) 1.0897 (0.0926)	0.5581 (0.0143) 1.1452 (0.1210)	(0.3349, 0.7160) (0.5691, 1.6104)	0.3811/96.2 1.0413/94.4
	${S}_{3}$	$\alpha$ $\beta$	0.5221 (0.0082) 1.0857 (0.0872)	0.5726 (0.0148) 1.2460 (0.1820)	(0.3519, 0.6923) (0.5855, 1.5859)	0.3404/95.3 1.0004/94.0
	${S}_{4}$	$\alpha$ $\beta$	0.5289 (0.0108) 1.1177 (0.1326)	0.5573 (0.0145) 1.1732 (0.1701)	(0.3349, 0.7230) (0.5089, 1.7265)	0.3881/95.9 1.2175/94.5
(80, 60)	${S}_{1}$	$\alpha$ $\beta$	0.5198 (0.0080) 1.0470 (0.0404)	0.5634 (0.0130) 1.1412 (0.0687)	(0.3562, 0.3562) (0.6992, 1.3948)	0.3271/95.4 0.6955/94.8
	${S}_{2}$	$\alpha$ $\beta$	0.5212 (0.0081) 1.0550 (0.0475)	0.5477 (0.0107) 1.0968 (0.0590)	(0.3573, 0.6851) (0.6815, 1.4284)	0.3278/95.5 0.7469/94.5
	${S}_{3}$	$\alpha$ $\beta$	0.5197 (0.0073) 1.0541 (0.0469)	0.5600 (0.0116) 1.1545 (0.0807)	(0.3634, 0.6760) (0.6823, 1.4259)	0.3125/95.8 0.7436/94.6
	${S}_{4}$	$\alpha$ $\beta$	0.5223 (0.0082) 1.0625 (0.0549)	0.5459 (0.0105) 1.0993 (0.0662)	(0.3579, 0.6866) (0.6642, 1.4609)	0.3287/95.5 0.7967/94.1

Sch.—scheme, Parm.—Parameter.

**Table 4:** Avg., MSE (in practices), Asy-CI, AILs and CPs (in %) for Non-BEs of the Kum. distribution for different PCS-II when $\alpha =1$ , $\beta =1$ at different values of $n$ and $m$.

$(n,m)$	Sch	Parm	Non-Bayesian		Asy CI	
			MLE	MPS	(Lower, Upper)	AIL/CP
(40, 20)	${S}_{1}$	$\alpha$ $\beta$	1.0723 (0.0811) 1.1375 (0.1923)	1.2935 (0.2098) 1.4239 (0.5218)	(0.5489, 1.5958) (0.4748, 1.8002)	1.0469/95.2 1.3253/93.5
	${S}_{2}$	$\alpha$ $\beta$	1.0951 (0.0950) 1.2076 (0.3543)	1.2114 (0.1559) 1.3238 (0.5547)	(0.5471, 1.6430) (0.3564, 2.0588)	1.0959/94.9 1.7024/92.7
	${S}_{3}$	$\alpha$ $\beta$	1.0855 (0.0798) 1.2023 (0.3478)	1.2710 (0.1753) 1.5564 (0.9634)	(0.5912, 1.5798) (0.3895, 2.0150)	0.9886/95.0 1.6254/92.8
	${S}_{4}$	$\alpha$ $\beta$	1.1120 (0.1079) 1.2811 (0.5882)	1.2126 (0.1621) 1.4027 (0.9066)	(0.5427, 1.6812) (0.2292, 2.3331)	1.1384/94.5 2.1038/92.4
(40, 30)	${S}_{1}$	$\alpha$ $\beta$	1.0826 (0.0733) 1.1029 (0.1068)	1.2405 (0.1471) 1.2881 (0.2362)	(0.6117, 1.5535) (0.5775, 1.6283)	0.9418/95.3 1.0507/93.4
	${S}_{2}$	$\alpha$ $\beta$	1.0899 (0.0775) 1.1219 (0.1328)	1.1858 (0.1169) 1.2043 (0.1871)	(0.6131, 1.5667) (0.5488, 1.6950)	0.9536/95.2 1.1462/93.3
	${S}_{3}$	$\alpha$ $\beta$	1.0854 (0.0723) 1.1214 (0.1327)	1.2324 (0.1378) 1.3223 (0.2971)	(0.6291, 1.5417) (0.5515, 1.6913)	0.9125/94.9 1.1398/93.3
	${S}_{4}$	$\alpha$ $\beta$	1.0959 (0.0813) 1.1405 (0.1615)	1.1801 (0.1157) 1.2111 (0.2156)	(0.6135, 1.5782) (0.5197, 1.7613)	0.9646/95.0 1.2415/93.6
(80, 40)	${S}_{1}$	$\alpha$ $\beta$	1.0409 (0.0370) 1.0608 (0.0594)	1.1638 (0.0737) 1.1992 (0.1207)	(0.6663, 1.4155) (0.6328, 1.4887)	0.7491/96.3 0.8559/94.7
	${S}_{2}$	$\alpha$ $\beta$	1.0509 (0.0403) 1.0897 (0.0926)	1.1163 (0.0573) 1.1451 (0.1210)	(0.6698, 1.4321) (0.5691, 1.6104)	0.7622/96.2 1.0413/94.4
	${S}_{3}$	$\alpha$ $\beta$	1.0443 (0.0331) 1.0857 (0.0872)	1.1453 (0.0593) 1.2460 (0.1819)	(0.7039, 1.3847) (0.5855, 1.5859)	0.6808/95.3 1.0004/94.0
	${S}_{4}$	$\alpha$ $\beta$	1.0579 (0.0434) 1.1177 (0.1326)	1.1148 (0.0583) 1.1733 (0.1702)	(0.6698, 1.4460) (0.5089, 1.7265)	0.7762/95.9 1.2175/94.5
(80, 60)	${S}_{1}$	$\alpha$ $\beta$	1.0397 (0.0320) 1.0470 (0.0404)	1.1269 (0.0521) 1.1412 (0.0687)	(0.7125, 1.3668) (0.6992, 1.3948)	0.6542/95.4 0.6956/94.8
	${S}_{2}$	$\alpha$ $\beta$	1.0424 (0.0326) 1.0550 (0.0475)	1.0956 (0.0430) 1.0969 (0.0590)	(0.7146, 1.3703) (0.6815, 1.4284)	0.6557/95.5 0.7469/94.5
	${S}_{3}$	$\alpha$ $\beta$	1.0394 (0.0294) 1.0541 (0.0469)	1.1200 (0.0465) 1.1545 (0.0807)	(0.7269, 1.3520) (0.6823, 1.4259)	0.6251/95.8 0.7436/94.7
	${S}_{4}$	$\alpha$ $\beta$	1.0445 (0.0331) 1.0625 (0.0549)	1.0918 (0.0422) 1.0992 (0.0662)	(0.7158, 1.3733) (0.6642, 1.4609)	0.6575/95.5 0.7967/94.1

Sch.—scheme, Parm.—Parameter.

**Table 5:** Avg., MSE (in practices), Asy-CI, AILs and CPs (in %) for Non-BEs of the Kum. distribution for different PCS-II when $\alpha =1$ , $\beta =2$ at different values of $n$ and $m$.

$(n,m)$	Sch	Parm	Non-Bayesian		Asy CI	
			MLE	MPS	(Lower, Upper)	AIL/CP
(40, 20)	${S}_{1}$	$\alpha$ $\beta$	1.0611 (0.0605) 2.3592 (1.2109)	1.2454 (0.1456) 3.1189 (3.7435)	(0.6096, 1.5127) (0.7664, 3.9519)	0.9031/95.3 3.1854/93.0
	${S}_{2}$	$\alpha$ $\beta$	1.0831 (0.0751) 2.5807 (2.6560)	1.1776 (0.1162) 2.9212 (4.6127)	(0.5938, 1.5724) (0.3450, 4.8165)	0.9785/95.3 4.4715/92.8
	${S}_{3}$	$\alpha$ $\beta$	1.0728 (0.0600) 2.5341 (2.3793)	1.2339 (0.1298) 3.4893 (7.5024)	(0.6442, 1.5014) (0.5346, 4.5336)	0.8571/95.3 3.9989/92.9
	${S}_{4}$	$\alpha$ $\beta$	1.0994 (0.0877) 2.8139 (4.9418)	1.1838 (0.1269) 3.1945 (8.5153)	(0.5831, 1.6158) (0.0000, 5.7260)	1.0327/94.9 5.7260/93.0
(40, 30)	${S}_{1}$	$\alpha$ $\beta$	1.0671 (0.0521) 2.2708 (0.6499)	1.1989 (0.1006) 2.7501 (1.5818)	(0.6680, 1.4662) (1.0188, 3.5229)	0.7981/95.3 2.5040/93.1
	${S}_{2}$	$\alpha$ $\beta$	1.0749 (0.0570) 2.3317 (0.8764)	1.1500 (0.0812) 2.5467 (1.2949)	(0.6627, 1.4871) (0.9132, 3.7502)	0.8243/95.2 2.8369/93.4
	${S}_{3}$	$\alpha$ $\beta$	1.0697 (0.0517) 2.3199 (0.8370)	1.1948 (0.0966) 2.8430 (2.0558)	(0.6813, 1.4581) (0.9488, 3.6909)	0.7767/94.7 2.7421/93.2
	${S}_{4}$	$\alpha$ $\beta$	1.0810 (0.0610) 2.3891 (1.1277)	1.1477 (0.0830) 2.5802 (1.5758)	(0.6590, 1.5029) (0.8135, 3.9646)	0.8439/95.1 3.1511/93.1
(80, 40)	${S}_{1}$	$\alpha$ $\beta$	1.0347 (0.0276) 2.1571 (0.3308)	1.1382 (0.0528) 2.5070 (0.7435)	(0.7122, 1.3573) (1.1595, 3.1547)	0.6451/96.3 1.9951/94.5
	${S}_{2}$	$\alpha$ $\beta$	1.0447 (0.0321) 2.2443 (0.5916)	1.0980 (0.0438) 2.3929 (0.8114)	(0.7040, 1.3854) (0.9515, 3.5370)	0.6813/96.0 2.5854/94.6
	${S}_{3}$	$\alpha$ $\beta$	1.0377 (0.0252) 2.2197 (0.5071)	1.1258 (0.0445) 2.6213 (1.1478)	(0.7423, 1.3331) (1.0466, 3.3928)	0.5907/95.5 2.3461/94.0
	${S}_{4}$	$\alpha$ $\beta$	1.0516 (0.0356) 2.3257 (0.9068)	1.0995 (0.0466) 2.4799 (1.2175)	(0.6989, 1.4043) (0.7716, 3.8797)	0.7054/96.1 3.1081/94.7
(80, 60)	${S}_{1}$	$\alpha$ $\beta$	1.0317 (0.0229) 2.1209 (0.2241)	1.1053 (0.0363) 2.3560 (0.4091)	(0.7535, 1.3100) (1.3121, 2.9297)	0.5564/95.9 1.6176/94.2
	${S}_{2}$	$\alpha$ $\beta$	1.0350 (0.0242) 2.1457 (0.2801)	1.0768 (0.0307) 2.2506 (0.3588)	(0.7503, 1.3197) (1.2509, 3.0405)	0.5693/95.9 1.7895/94.3
	${S}_{3}$	$\alpha$ $\beta$	1.0319 (0.0213) 2.1386 (0.2629)	1.1012 (0.0332) 2.3888 (0.4833)	(0.7646, 1.2993) (1.2703, 3.0068)	0.5347/95.9 1.7365/94.6
	${S}_{4}$	$\alpha$ $\beta$	1.0374 (0.0252) 2.1679 (0.3358)	1.0751 (0.0311) 2.2625 (0.4168)	(0.7483, 1.3264) (1.1963, 3.1395)	0.5780/95.8 1.9431/94.2

Sch.—scheme, Parm.—Parameter.

Tables 6, 7, 8, 9, 10, 11, 12, 13 show BE obtained using Lindley's approximation and MCMC method with different loss functions, for various values of $n$ and $m$. The first column of the tables indicates the average estimates (Avg.), while the second column represents the mean square errors (MSEs). In Tables 14, 15, we present confidence intervals, which include the highest posterior density (HPD) intervals, average interval length (AIL), and coverage probability (CP) using the MCMC method.

**Table 6:** Avg. and MSE (in practices) for BEs (Lindely) of the Kum. distribution for different PCS-II when $\alpha =0.5$ , $\beta =0.5$ at different values of $n$ and $m$.

$(n,m)$	Sch	Parm	BEs: Lindely				
			SE	LINEX		GE	
				$c =-0.5$	$c=1.5$	$q=0.1$	$q=2$
(40, 20)	${S}_{1}$	$\alpha$ $\beta$	0.9384 (0.2621) 0.2434 (0.1019)	0.9052 (0.2228) 0.2074 (0.1350)	1.1104 (0.5499) 0.3055 (0.0641)	1.1433 (0.5670) 0.3311 (0.0551)	1.7933 (4.3371) 0.3840 (0.0341)
	${S}_{2}$	$\alpha$ $\beta$	1.0057 (0.3796) 0.1631 (0.1948)	0.9586 (0.3037) 0.1002 (0.3235)	1.1693 (0.6927) 0.2771 (0.0637)	1.3104 (1.1592) 0.2991 (0.0508)	1.7606 (3.7712) 0.3706 (0.0307)
	${S}_{3}$	$\alpha$ $\beta$	1.0468 (0.4476) 0.0926 (0.3341)	0.9884 (0.3427) 0.1800 (0.1189)	1.2518 (0.9280) 0.2457 (0.0754)	1.4557 (1.7338) 0.2740 (0.0589)	1.5312 (1.8767) 0.3588 (0.0341)
	${S}_{4}$	$\alpha$ $\beta$	0.8339 (0.1952) 0.2785 (0.1088)	0.8173 (0.1731) 0.2481 (0.1445)	0.9009 (0.3202) 0.3560 (0.0371)	0.9118 (0.3171) 0.3608 (0.0313)	1.2634 (1.3975) 0.4121 (0.0274)
(40, 30)	${S}_{1}$	$\alpha$ $\beta$	0.7872 (0.1402) 0.3823 (0.0494)	0.7725 (0.1279) 0.3702 (0.0547)	0.8584 (0.2580) 0.4074 (0.0434)	0.8620 (0.2319) 0.4201 (0.0469)	1.0808 (1.1538) 0.4432 (0.0476)
	${S}_{2}$	$\alpha$ $\beta$	0.8390 (0.2043) 0.3512 (0.0516)	0.8161 (0.1751) 0.3314 (0.0660)	0.9288 (0.3767) 0.3851 (0.0387)	0.9682 (0.4341) 0.3983 (0.0407)	1.1200 (1.5211) 0.4248 (0.0260)
	${S}_{3}$	$\alpha$ $\beta$	0.8252 (0.1851) 0.3583 (0.0480)	0.8049 (0.1601) 0.3399 (0.0605)	0.9067 (0.3434) 0.3897 (0.0363)	0.9369 (0.3672) 0.4018 (0.0377)	1.1331 (1.6838) 0.4391 (0.0813)
	${S}_{4}$	$\alpha$ $\beta$	0.7530 (0.1212) 0.3960 (0.0362)	0.7432 (0.1111) 0.3849 (0.0410)	0.7943 (0.1864) 0.4164 (0.0308)	0.7992 (0.1773) 0.4245 (0.0311)	0.9653 (0.9463) 0.4483 (0.0453)
(80, 40)	${S}_{1}$	$\alpha$ $\beta$	0.6868 (0.0518) 0.3933 (0.0215)	0.6829 (0.0502) 0.3901 (0.0224)	0.7004 (0.0578) 0.4014 (0.0195)	0.0224 (0.0582) 0.4058 (0.0181)	0.7587 (0.0848) 0.4199 (0.0151)
	${S}_{2}$	$\alpha$ $\beta$	0.6751 (0.0493) 0.4103 (0.0171)	0.6718 (0.0477) 0.4075 (0.0175)	0.6871 (0.0554) 0.4172 (0.0164)	0.6894 (0.0554) 0.4201 (0.0155)	0.7349 (0.0820) 0.4318 (0.0141)
	${S}_{3}$	$\alpha$ $\beta$	0.6572 (0.0418) 0.4117 (0.0157)	0.6545 (0.0406) 0.4090 (0.0159)	0.6667 (0.0466) 0.4184 (0.0152)	0.6683 (0.0466) 0.4210 (0.0145)	0.7014 (0.0658) 0.4324 (0.0135)
	${S}_{4}$	$\alpha$ $\beta$	0.6139 (0.0297) 0.4555 (0.0142)	0.6138 (0.0296) 0.4551 (0.0141)	0.6141 (0.0301) 0.4567 (0.0145)	0.6136 (0.0300) 0.4573 (0.0142)	0.6119 (0.0308) 0.4604 (0.0143)
(80, 60)	${S}_{1}$	$\alpha$ $\beta$	0.6141 (0.0261) 0.4491 (0.0124)	0.6140 (0.0261) 0.4485 (0.0127)	0.6145 (0.0262) 0.4507 (0.0118)	0.6144 (0.0262) 0.4520 (0.0114)	0.6148 (0.0271) 0.4554 (0.0103)
	${S}_{2}$	$\alpha$ $\beta$	0.6237 (0.0306) 0.4482 (0.0113)	0.6228 (0.0302) 0.4475 (0.0115)	0.6265 (0.0321) 0.4499 (0.0109)	0.6268 (0.0321) 0.4510 (0.0106)	0.6363 (0.0383) 0.4546 (0.0097)
	${S}_{3}$	$\alpha$ $\beta$	0.6308 (0.0332) 0.4418 (0.0113)	0.6293 (0.0325) 0.4408 (0.0115)	0.6363 (0.0362) 0.4444 (0.0108)	0.6371 (0.0362) 0.4458 (0.0105)	0.6594 (0.0534) 0.4507 (0.0096)
	${S}_{4}$	$\alpha$ $\beta$	0.6868 (0.0518) 0.3933 (0.0215)	0.6829 (0.0502) 0.3901 (0.0224)	0.7004 (0.0578) 0.4014 (0.0195)	0.0224 (0.0582) 0.4058 (0.0181)	0.7587 (0.0848) 0.4199 (0.0151)

Sch.—scheme, Parm.—Parameter.

**Table 7:** Avg. and MSE (in practices) for BEs (Lindely) of the Kum. distribution for different PCS-II when $\alpha =0.5$ , $\beta =1$ at different values of $n$ and $m$.

$(n,m)$	Sch	Parm	BEs: Lindely				
			SE	LINEX		GE	
				$c =-0.5$	$c=1.5$	$q=0.1$	$q=2$
(40, 20)	${S}_{1}$	$\alpha$ $\beta$	0.7431 (0.0908) 0.6334 (0.2351)	0.7344 (0.0845) 0.5416 (0.3844)	0.7814 (0.1305) 0.7596 (0.1607)	0.7861 (0.1230) 0.7367 (0.1468)	1.1019 (1.0265) 0.8124 (0.1169)
	${S}_{2}$	$\alpha$ $\beta$	0.7825 (0.1399) 0.4621 (0.7862)	0.7687 (0.1239) 0.3887 (0.6555)	0.8397 (0.2394) 0.7525 (0.1706)	0.8503 (0.2378) 0.6844 (0.1413)	1.1399 (1.1512) 0.8059 (0.1153)
	${S}_{3}$	$\alpha$ $\beta$	0.8107 (0.1644) 0.3170 (1.5505)	0.7918 (0.1404) 0.2331 (1.0237)	0.8844 (0.2830) 0.7013 (0.1832)	0.9130 (0.3627) 0.6244 (0.1700)	1.3018 (1.5354) 0.7700 (0.1145)
	${S}_{4}$	$\alpha$ $\beta$	0.7083 (0.0947) 0.6350 (0.7184)	0.7027 (0.0880) 0.5937 (0.5030)	0.7262 (0.1205) 0.8823 (0.2197)	0.7310 (0.1245) 0.8039 (0.1057)	0.8291 (0.5578) 0.8989 (0.1452)
(40, 30)	${S}_{1}$	$\alpha$ $\beta$	0.6615 (0.0499) 0.8447 (0.1231)	0.6587 (0.0480) 0.8175 (0.1439)	0.6737 (0.0604) 0.8857 (0.1129)	0.6743 (0.0587) 0.8784 (0.1075)	0.7517 (0.2581) 0.9084 (0.0995)
	${S}_{2}$	$\alpha$ $\beta$	0.6888 (0.0718) 0.7903 (0.1214)	0.6834 (0.0669) 0.7125 (0.3999)	0.7155 (0.1092) 0.8604 (0.0973)	0.7131 (0.0955) 0.8443 (0.0944)	0.7888 (0.2307) 0.8890 (0.0864)
	${S}_{3}$	$\alpha$ $\beta$	0.6853 (0.0684) 0.7900 (0.1176)	0.6801 (0.0638) 0.7242 (0.2617)	0.7084 (0.0963) 0.8589 (0.0944)	0.7090 (0.0905) 0.8429 (0.0917)	0.8146 (0.3849) 0.8860 (0.0824)
	${S}_{4}$	$\alpha$ $\beta$	0.6538 (0.0530) 0.8607 (0.0986)	0.6515 (0.0509) 0.8072 (0.2462)	0.6656 (0.0716) 0.9066 (0.0982)	0.6632 (0.0617) 0.8944 (0.0904)	0.7069 (0.1565) 0.9230 (0.0886)
(80, 40)	${S}_{1}$	$\alpha$ $\beta$	0.6050 (0.0206) 0.8655 (0.0642)	0.6045 (0.0205) 0.8623 (0.0657)	0.6062 (0.0207) 0.8746 (0.0609)	0.6064 (0.0207) 0.8735 (0.0605)	0.6082 (0.0208) 0.8850 (0.0564)
	${S}_{2}$	$\alpha$ $\beta$	0.6010 (0.0216) 0.8936 (0.0618)	0.6005 (0.0215) 0.8893 (0.0604)	0.6024 (0.0222) 0.9037 (0.0647)	0.6026 (0.0222) 0.9007 (0.0621)	0.6053 (0.0234) 0.9114 (0.0626)
	${S}_{3}$	$\alpha$ $\beta$	0.5930 (0.0186) 0.8852 (0.0579)	0.5926 (0.0184) 0.8804 (0.0565)	0.5945 (0.0191) 0.8965 (0.0609)	0.5946 (0.0191) 0.8931 (0.0584)	0.5975 (0.0204) 0.9048 (0.0591)
	${S}_{4}$	$\alpha$ $\beta$	0.5756 (0.0176) 0.9651 (0.0726)	0.5759 (0.0176) 0.9652 (0.0696)	0.5749 (0.0177) 0.9647 (0.0788)	0.5743 (0.0176) 0.9639 (0.0752)	0.5714 (0.0176) 0.9643 (0.0787)
(80, 60)	${S}_{1}$	$\alpha$ $\beta$	0.5659 (0.0119) 0.9425 (0.0439)	0.5663 (0.0120) 0.9437 (0.0447)	0.5645 (0.0116) 0.9397 (0.0420)	0.5638 (0.0116) 0.9412 (0.0425)	0.5593 (0.0110) 0.9397 (0.0405)
	${S}_{2}$	$\alpha$ $\beta$	0.5706 (0.0135) 0.9429 (0.0435)	0.5708 (0.0135) 0.9437 (0.0440)	0.5699 (0.0134) 0.9411 (0.0425)	0.5694 (0.0134) 0.9420 (0.0426)	0.5668 (0.0133) 0.9415 (0.0415)
	${S}_{3}$	$\alpha$ $\beta$	0.5744 (0.0140) 0.9277 (0.0416)	0.5743 (0.0139) 0.9273 (0.0419)	0.5745 (0.0141) 0.9288 (0.0410)	0.5743 (0.0141) 0.9288 (0.0408)	0.5740 (0.0146) 0.9310 (0.0399)
	${S}_{4}$	$\alpha$ $\beta$	0.5615 (0.0121) 0.9623 (0.0468)	0.5619 (0.0122) 0.9641 (0.0472)	0.5603 (0.0120) 0.9581 (0.0459)	0.5597 (0.0119) 0.9597 (0.0461)	0.5559 (0.0116) 0.9567 (0.0453)

Sch.—scheme, Parm.—Parameter.

**Table 8:** Avg. and MSE (in practices) for BEs (Lindely) of the Kum. distribution for different PCS-II when $\alpha =1$ , $\beta =1$ at different values of $n$ and $m$.

$(n,m)$	Sch	Parm	BEs: Lindely				
			SE	LINEX		GE	
				$c =-0.5$	$c=1.5$	$q=0.1$	$q=2$
(40, 20)	${S}_{1}$	$\alpha$ $\beta$	1.4863 (0.3634) 0.6334 (0.2351)	1.4540 (0.3183) 0.5416 (0.3843)	1.6294 (0.6102) 0.7596 (0.1607)	1.5722 (0.4921) 0.7367 (0.1468)	2.2041 (4.1173) 0.8124 (0.1169)
	${S}_{2}$	$\alpha$ $\beta$	1.5650 (0.5598) 0.4620 (0.7863)	1.5151 (0.4516) 0.3886 (0.6555)	1.6790 (0.7999) 0.7525 (0.1706)	1.7007 (0.9515) 0.6844 (0.1413)	2.2808 (4.6401) 0.8059 (0.1153)
	${S}_{3}$	$\alpha$ $\beta$	1.6215 (0.6579) 0.3170 (1.5511)	1.5542 (0.4994) 0.2332 (1.0227)	1.7666 (0.9429) 0.7013 (0.1832)	1.8261 (1.4509) 0.6243 (0.1700)	2.6023 (6.0872) 0.7700 (0.1145)
	${S}_{4}$	$\alpha$ $\beta$	1.4167 (0.3791) 0.6350 (0.7179)	1.3960 (0.3328) 0.5940 (0.4963)	1.4498 (0.4554) 0.8823 (0.2196)	1.4620 (0.4983) 0.8039 (0.1057)	1.6586 (2.2416) 0.8989 (0.1452)
(40, 30)	${S}_{1}$	$\alpha$ $\beta$	1.3231 (0.1999) 0.8447 (0.1231)	1.3123 (0.1857) 0.8175 (0.1439)	1.3707 (0.2905) 0.8857 (0.1129)	1.3487 (0.2348) 0.8784 (0.1075)	1.5036 (1.0374) 0.9084 (0.0995)
	${S}_{2}$	$\alpha$ $\beta$	1.3776 (0.2874) 0.7903 (0.1214)	1.3578 (0.2527) 0.7125 (0.3994)	1.4303 (0.3958) 0.8605 (0.0973)	1.4262 (0.3820) 0.8443 (0.0944)	1.5778 (0.9234) 0.8890 (0.0864)
	${S}_{3}$	$\alpha$ $\beta$	1.3707 (0.2738) 0.7900 (0.1176)	1.3515 (0.2413) 0.7242 (0.2620)	1.4222 (0.3762) 0.8589 (0.0944)	1.4181 (0.3622) 0.8429 (0.0917)	1.6293 (1.5423) 0.8861 (0.0824)
	${S}_{4}$	$\alpha$ $\beta$	1.3076 (0.2122) 0.8607 (0.0986)	1.2989 (0.1968) 0.8073 (0.2461)	1.3346 (0.2764) 0.9066 (0.0982)	1.3264 (0.2468) 0.8944 (0.0904)	1.4138 (0.6259) 0.9230 (0.0886)
(80, 40)	${S}_{1}$	$\alpha$ $\beta$	1.2100 (0.0824) 0.8655 (0.0642)	1.2083 (0.0818) 0.8623 (0.0657)	1.2147 (0.0838) 0.8746 (0.0609)	1.2129 (0.0830) 0.8735 (0.0605)	1.2164 (0.0835) 0.8850 (0.0564)
	${S}_{2}$	$\alpha$ $\beta$	1.2020 (0.0867) 0.8936 (0.0618)	1.2002 (0.0855) 0.8893 (0.0604)	1.2080 (0.0917) 0.9037 (0.0647)	1.2052 (0.0888) 0.9007 (0.0621)	1.2107 (0.0936) 0.9114 (0.0626)
	${S}_{3}$	$\alpha$ $\beta$	1.1861 (0.0745) 0.8852 (0.0579)	1.1844 (0.0733) 0.8804 (0.0565)	1.1921 (0.0794) 0.8965 (0.0609)	1.1893 (0.0767) 0.8931 (0.0584)	1.1950 (0.0818) 0.9048 (0.0591)
	${S}_{4}$	$\alpha$ $\beta$	1.1513 (0.0707) 0.9651 (0.0726)	1.1522 (0.0707) 0.9652 (0.0696)	1.1481 (0.0711) 0.9647 (0.0788)	1.1487 (0.0707) 0.9639 (0.0752)	1.1428 (0.0707) 0.9643 (0.0787)
(80, 60)	${S}_{1}$	$\alpha$ $\beta$	1.1318 (0.0477) 0.9425 (0.0439)	1.1335 (0.0484) 0.9437 (0.0447)	1.1257 (0.0456) 0.9397 (0.0420)	1.1277 (0.0465) 0.9412 (0.0425)	1.1187 (0.0441) 0.9397 (0.0405)
	${S}_{2}$	$\alpha$ $\beta$	1.1412 (0.0541) 0.9429 (0.0435)	1.1420 (0.0543) 0.9437 (0.0440)	1.1381 (0.0536) 0.9411 (0.0425)	1.1389 (0.0538) 0.9420 (0.0426)	1.1336 (0.0532) 0.9415 (0.0415)
	${S}_{3}$	$\alpha$ $\beta$	1.1488 (0.0560) 0.9277 (0.0416)	1.1486 (0.0556) 0.9273 (0.0419)	1.1492 (0.0576) 0.9288 (0.0410)	1.1486 (0.0567) 0.9288 (0.0408)	1.1480 (0.0586) 0.9310 (0.0399)
	${S}_{4}$	$\alpha$ $\beta$	1.1231 (0.0486) 0.9623 (0.0468)	1.1245 (0.0489) 0.9641 (0.0472)	1.1179 (0.0473) 0.9581 (0.0459)	1.1195 (0.0478) 0.9597 (0.0461)	1.1119 (0.0465) 0.9567 (0.0453)

Sch.—scheme, Parm.—Parameter.

**Table 9:** Avg. and MSE (in practices) for BEs (Lindely) of the Kum. distribution for different PCS-II when $\alpha =1$ , $\beta =2$ at different values of $n$ and $m$.

$(n,m)$	Sch	Parm	BEs: Lindely				
			SE	LINEX		GE	
				$c =-0.5$	$c=1.5$	$q=0.1$	$q=2$
(40, 20)	${S}_{1}$	$\alpha$ $\beta$	1.2953 (0.1670) 1.5058 (0.6729)	1.2865 (0.1574) 1.3338 (0.8986)	1.3228 (0.1972) 1.7729 (0.7509)	1.3151 (0.1882) 1.6374 (0.5063)	1.3856 (0.4637) 1.7475 (0.5341)
	${S}_{2}$	$\alpha$ $\beta$	1.3517 (0.2760) 1.1394 (4.8587)	1.3344 (0.2422) 1.0818 (1.4288)	1.3855 (0.3594) 1.8918 (1.3713)	1.3913 (0.3697) 1.5640 (0.4520)	1.5128 (0.9525) 1.7789 (0.6130)
	${S}_{3}$	$\alpha$ $\beta$	1.3815 (0.3014) 0.8658 (9.7304)	1.3572 (0.2530) 0.8354 (2.0282)	1.4467 (0.4523) 1.7984 (1.2275)	1.4430 (0.4776) 1.4322 (0.4894)	1.6143 (1.1835) 1.6864 (0.5185)
	${S}_{4}$	$\alpha$ $\beta$	1.2905 (0.2365) 1.3617 (7.9165)	1.2816 (0.2152) 1.4465 (0.6741)	1.2987 (0.2678) 2.1645 (2.7792)	1.3078 (0.2856) 1.7988 (0.5129)	1.3481 (0.5208) 1.9952 (1.0520)
(40, 30)	${S}_{1}$	$\alpha$ $\beta$	1.2029 (0.0991) 1.8345 (0.4101)	1.2013 (0.0967) 1.7695 (0.4771)	1.2120 (0.1184) 1.8921 (0.4416)	1.2058 (0.1037) 1.8626 (0.4024)	1.2161 (0.1238) 1.8925 (0.4116)
	${S}_{2}$	$\alpha$ $\beta$	1.2358 (0.1395) 1.7462 (0.3719)	1.2309 (0.1314) 1.6400 (0.6457)	1.2544 (0.1827) 1.9001 (0.5003)	1.2459 (0.1566) 1.8163 (0.3499)	1.2961 (0.3837) 1.8762 (0.3899)
	${S}_{3}$	$\alpha$ $\beta$	1.2333 (0.1341) 1.7248 (0.3579)	1.2279 (0.1259) 1.6188 (0.6318)	1.2536 (0.1788) 1.8821 (0.4666)	1.2443 (0.1518) 1.7971 (0.3308)	1.3081 (0.8800) 1.8590 (0.3681)
	${S}_{4}$	$\alpha$ $\beta$	1.2091 (0.1241) 1.8561 (0.3902)	1.2068 (0.1192) 1.7905 (0.4526)	1.2173 (0.1487) 1.9772 (0.6198)	1.2130 (0.1336) 1.9079 (0.4068)	1.2337 (0.2409) 1.9515 (0.4792)
(80, 40)	${S}_{1}$	$\alpha$ $\beta$	1.1293 (0.0438) 1.8559 (0.2654)	1.1301 (0.0441) 1.8640 (0.2709)	1.1260 (0.0425) 1.8526 (0.2619)	1.1271 (0.0430) 1.8538 (0.2618)	1.1214 (0.0414) 1.8556 (0.2573)
	${S}_{2}$	$\alpha$ $\beta$	1.1304 (0.0514) 1.9196 (0.3278)	1.1307 (0.0513) 1.9126 (0.2984)	1.1290 (0.0517) 1.9276 (0.3772)	1.1292 (0.0514) 1.9197 (0.3392)	1.1264 (0.0515) 1.9241 (0.3544)
	${S}_{3}$	$\alpha$ $\beta$	1.1204 (0.0423) 1.8882 (0.2881)	1.1206 (0.0422) 1.8834 (0.2659)	1.1195 (0.0427) 1.8991 (0.3284)	1.1196 (0.0424) 1.8897 (0.2977)	1.1175 (0.0427) 1.8961 (0.3103)
	${S}_{4}$	$\alpha$ $\beta$	1.1106 (0.0509) 2.0439 (0.4595)	1.1115 (0.0509) 2.0377 (0.4067)	1.1078 (0.0510) 2.0370 (0.5533)	1.1085 (0.0509) 2.0368 (0.4810)	1.1041 (0.0510) 2.0304 (0.5102)
(80, 60)	${S}_{1}$	$\alpha$ $\beta$	1.0840 (0.0285) 1.9581 (0.2009)	1.0855 (0.0288) 1.9705 (0.2072)	1.0791 (0.0275) 1.9329 (0.1902)	1.0806 (0.0279) 1.9485 (0.1973)	1.0739 (0.0268) 1.9361 (0.1926)
	${S}_{2}$	$\alpha$ $\beta$	1.0902 (0.0322) 1.9674 (0.2208)	1.0913 (0.0324) 1.9786 (0.2252)	1.0865 (0.0315) 1.9462 (0.2159)	1.0876 (0.0318) 1.9589 (0.2188)	1.0824 (0.0311) 1.9484 (0.2165)
	${S}_{3}$	$\alpha$ $\beta$	1.0931 (0.0310) 1.9364 (0.1987)	1.0939 (0.0311) 1.9443 (0.1999)	1.0905 (0.0307) 1.9231 (0.1995)	1.0912 (0.0308) 1.9307 (0.1983)	1.0874 (0.0304) 1.9244 (0.1981)
	${S}_{4}$	$\alpha$ $\beta$	1.0840 (0.0319) 2.0016 (0.2570)	1.0852 (0.0321) 2.0146 (0.2628)	1.0802 (0.0312) 1.9761 (0.2507)	1.0814 (0.0315) 1.9914 (0.2546)	1.0762 (0.0309) 1.9784 (0.2521)

Sch.—scheme, Parm.—Parameter.

**Table 10:** Avg. and MSE (in practices for BEs (MCMC) of the Kum. distribution for different PCS-II when $\alpha =0.5$ , $\beta =0.5$ at different values of $n$ and $m$.

$(n,m)$	Sch	Parm	BEs: MCMC					
			SE	LINEX		GE		
				$c =-0.5$	$c=1.5$	$q=0.1$		$q=2$
(40, 20)	${S}_{1}$	$\alpha$ $\beta$	0.4862 (0.0052) 0.4256 (0.0092)	0.4891 (0.0053) 0.4267 (0.0091)	0.4777 (0.0052) 0.4224 (0.0096)	0.4733 (0.0055) 0.4202 (0.0100)		0.4507 (0.0068) 0.4106 (0.0114)
	${S}_{2}$	$\alpha$ $\beta$	0.4680 (0.0048) 0.3928 (0.0134)	0.4704 (0.0048) 0.3937 (0.0132)	0.4609 (0.0051) 0.3903 (0.0139)	0.4569 (0.0055) 0.3881 (0.0144)		0.4373 (0.0072) 0.3800 (0.0162)
	${S}_{3}$	$\alpha$ $\beta$	0.4653 (0.0043) 0.3806 (0.0157)	0.4674 (0.0042) 0.3814 (0.0156)	0.4592 (0.0046) 0.3784 (0.0162)	0.4557 (0.0049) 0.3764 (0.0167)		0.4388 (0.0065) 0.3690 (0.0185)
	${S}_{4}$	$\alpha$ $\beta$	0.4766 (0.0050) 0.4205 (0.0094)	0.4792 (0.0050) 0.4218 (0.0093)	0.4690 (0.0051) 0.4167 (0.0099)	0.4648 (0.0054) 0.4141 (0.0104)		0.4389 (00,702) 0.4028 (0.0123)
(40, 30)	${S}_{1}$	$\alpha$ $\beta$	0.5026 (0.0041) 0.4649 (0.0039)	0.5049 (0.0041) 0.4657 (0.0039)	0.4959 (0.0038) 0.4627 (0.0041)	0.4927 (0.0039) 0.4615 (0.0041)		0.4754 (0.0042) 0.4556 (0.0046)
	${S}_{2}$	$\alpha$ $\beta$	0.4988 (0.0034) 0.4551 (0.0039)	0.5008 (0.0035) 0.4557 (0.0038)	0.4927 (0.0033) 0.4532 (0.0040)	0.4899 (0.0034) 0.4521 (0.0041)		0.4743 (0.0037) 0.4469 (0.0046)
	${S}_{3}$	$\alpha$ $\beta$	0.4946 (0.0032) 0.4514 (0.0042)	0.4966 (0.0032) 0.4520 (0.0042)	0.4890 (0.0031) 0.4495 (0.0044)	0.4862 (0.0032) 0.4483 (0.0045)		0.4714 (0.0037) 0.4430 (0.0051)
	${S}_{4}$	$\alpha$ $\beta$	0.5054 (0.0037) 0.4683 (0.0035)	0.5075 (0.0038) 0.4691 (0.0035)	0.4992 (0.0035) 0.4660 (0.0036)	0.4963 (0.0035) 0.4647 (0.0037)		0.4804 (0.0037) 0.4585 (0.0041)
(80, 40)	${S}_{1}$	$\alpha$ $\beta$	0.4985 (0.0057) 0.4673 (0.0056)	0.5008 (0.0058) 0.4683 (0.0056)	0.4918 (0.0055) 0.4646 (0.0057)	0.4887 (0.0056) 0.4632 (0.0058)		0.4715 (0.0060) 0.4559 (0.0062)
	${S}_{2}$	$\alpha$ $\beta$	0.4842 (0.0043) 0.4505 (0.0058)	0.4861 (0.0043) 0.4514 (0.0058)	0.4787 (0.0044) 0.4478 (0.0060)	0.4759 (0.0045) 0.4462 (0.4462)		0.4613 (0.0053) 0.4387 (0.0069)
	${S}_{3}$	$\alpha$ $\beta$	0.4871 (0.0041) 0.4536 (0.0057)	0.4888 (0.0041) 0.4545 (0.0056)	0.4821 (0.0041) 0.4507 (0.0059)	0.4795 (0.0042) 0.4491 (0.0060)		0.4662 (0.0048) 0.4413 (0.0067)
	${S}_{4}$	$\alpha$ $\beta$	0.4999 (0.0043) 0.4799 (0.0050)	0.5018 (0.0044) 0.4812 (0.0050)	0.4944 (0.0042) 0.4762 (0.0050)	0.4919 (0.0043) 0.4744 (0.0051)		0.4776 (0.0045) 0.4648 (0.0055)
(80, 60)	${S}_{1}$	$\alpha$ $\beta$	0.5069 (0.0046) 0.4885 (0.0033)	0.5085 (0.0046) 0.4891 (0.0033)	0.5019 (0.0044) 0.4867 (0.0032)	0.4997 (0.0044) 0.4858 (0.0033)		0.4872 (0.0044) 0.4813 (0.0033)
	${S}_{2}$	$\alpha$ $\beta$	0.5023 (0.0039) 0.4817 (0.0030)	0.5038 (0.0040) 0.4822 (0.0029)	0.4977 (0.0038) 0.4800 (0.0030)	0.4957 (0.0038) 0.4791 (0.0030)		0.4841 (0.0039) 0.4747 (0.0032)
	${S}_{3}$	$\alpha$ $\beta$	0.5005 (0.0034) 0.4775 (0.0028)	0.5018 (0.0034) 0.4781 (0.0028)	0.4964 (0.0033) 0.4759 (0.0028)	0.4945 (0.0033) 0.4751 (0.0029)		0.4841 (0.0034) 0.4709 (0.0031)
	${S}_{4}$	$\alpha$ $\beta$	0.5077 (0.0041) 0.4900 (0.0031)	0.5092 (0.0042) 0.4907 (0.0031)	0.5032 (0.0039) 0.4881 (0.0031)	0.5012 (0.0039) 0.4871 (0.0032)		0.4898 (0.0039) 0.4821 (0.0032)

Sch.—scheme, Parm.—Parameter.

**Table 11:** Avg. and MSE (in practices) for BEs (MCMC) of the Kum. distribution for different PCS-II when $\alpha =0.5$ , $\beta =1$ at different values of $n$ and $m$.

$(n,m)$	Sch	Parm	BEs: MCMC					
			SE	LINEX		GE		
				$c =-0.5$	$c=1.5$	$q=0.1$		$q=2$
(40, 20)	${S}_{1}$	$\alpha$ $\beta$	0.4876 (0.0036) 0.8779 (0.0334)	0.4895 (0.0036) 0.8836 (0.0326)	0.4819 (0.0036) 0.8615 (0.0361)	0.4790 (0.0038) 0.8643 (0.0362)		0.4640 (0.0045) 0.8408 (0.0419)
	${S}_{2}$	$\alpha$ $\beta$	0.4718 (0.0032) 0.8123 (0.0452)	0.4734 (0.0032) 0.8170 (0.0438)	0.4672 (0.0034) 0.7988 (0.0498)	0.4646 (0.0036) 0.8001 (0.0496)		0.4519 (0.0045) 0.7789 (0.0579)
	${S}_{3}$	$\alpha$ $\beta$	0.4705 (0.0029) 0.7884 (0.0527)	0.4719 (0.0028) 0.7924 (0.0512)	0.4666 (0.0031) 0.7768 (0.0573)	0.4643 (0.0032) 0.7776 (0.0571)		0.4534 (0.0040) 0.7589 (0.0654)
	${S}_{4}$	$\alpha$ $\beta$	0.4811 (0.0032) 0.8680 (0.0331)	0.4828 (0.0031) 0.8753 (0.0318)	0.4760 (0.0033) 0.8474 (0.0375)	0.4733 (0.0034) 0.8506 (0.0373)		0.4595 (0.0042) 0.8206 (0.0460)
(40, 30)	${S}_{1}$	$\alpha$ $\beta$	0.5015 (0.0025) 0.9470 (0.0162)	0.5029 (0.0026) 0.9508 (0.0161)	0.4973 (0.0025) 0.9359 (0.0168)	0.4953 (0.0025) 0.9385 (0.0169)		0.4844 (0.0026) 0.9237 (0.0184)
	${S}_{2}$	$\alpha$ $\beta$	0.4979 (0.0021) 0.9274 (0.0147)	0.4992 (0.0021) 0.9307 (0.0144)	0.4942 (0.0020) 0.9177 (0.0158)	0.4924 (0.0021) 0.9198 (0.0157)		0.4827 (0.0022) 0.9066 (0.0177)
	${S}_{3}$	$\alpha$ $\beta$	0.4942 (0.0020) 0.9180 (0.0161)	0.4954 (0.0020) 0.9213 (0.0157)	0.4907 (0.0020) 0.9083 (0.0174)	0.4889 (0.0020) 0.9103 (0.0173)		0.4797 (0.0022) 0.8969 (0.0195)
	${S}_{4}$	$\alpha$ $\beta$	0.5039 (0.0022) 0.9541 (0.0144)	0.5052 (0.0023) 0.9582 (0.0142)	0.5000 (0.0021) 0.9420 (0.0150)	0.4982 (0.0022) 0.9449 (0.0150)		0.4882 (0.0022) 0.9289 (0.0166)
(80, 40)	${S}_{1}$	$\alpha$ $\beta$	0.4987 (0.0038) 0.9540 (0.0242)	0.5003 (0.0039) 0.9589 (0.0244)	0.4943 (0.0037) 0.9399 (0.0242)	0.4921 (0.0038) 0.9433 (0.0247)		0.4806 (0.0040) 0.9249 (0.0259)
	${S}_{2}$	$\alpha$ $\beta$	0.4859 (0.0029) 0.9183 (0.0236)	0.4872 (0.0029) 0.9234 (0.0232)	0.4822 (0.0029) 0.9034 (0.0250)	0.4803 (0.0030) 0.9065 (0.0251)		0.4704 (0.0034) 0.8862 (0.0283)
	${S}_{3}$	$\alpha$ $\beta$	0.4908 (0.0027) 0.9294 (0.0230)	0.4919 (0.0027) 0.9346 (0.0227)	0.4875 (0.0027) 0.9143 (0.0240)	0.4858 (0.0027) 0.9176 (0.0242)		0.4771 (0.0030) 0.8972 (0.0269)
	${S}_{4}$	$\alpha$ $\beta$	0.4999 (0.0027) 0.9763 (0.0224)	0.5012 (0.0028) 0.9834 (0.0229)	0.4962 (0.0027) 0.9559 (0.0218)	0.4945 (0.0027) 0.9611 (0.0225)		0.4848 (0.0029) 0.9351 (0.0238)
(80, 60)	${S}_{1}$	$\alpha$ $\beta$	0.5049 (0.0029) 0.9881 (0.0151)	0.5060 (0.0029) 0.9913 (0.0153)	0.5017 (0.0028) 0.9790 (0.0147)	0.5003 (0.0028) 0.9814 (0.0150)		0.4922 (0.0028) 0.9698 (0.0151)
	${S}_{2}$	$\alpha$ $\beta$	0.5012 (0.0024) 0.9756 (0.0135)	0.5022 (0.0024) 0.9787 (0.0135)	0.4983 (0.0024) 0.9665 (0.0134)	0.4970 (0.0024) 0.9689 (0.0136)		0.4895 (0.0024) 0.9571 (0.0140)
	${S}_{3}$	$\alpha$ $\beta$	0.5001 (0.0021) 0.9681 (0.0123)	0.5010 (0.0021) 0.9710 (0.0123)	0.4975 (0.0020) 0.9597 (0.0125)	0.4963 (0.0020) 0.9618 (0.0125)		0.4897 (0.0021) 0.9509 (0.0132)
	${S}_{4}$	$\alpha$ $\beta$	0.5063 (0.0025) 0.9933 (0.0147)	0.5073 (0.0025) 0.9969 (0.0149)	0.5034 (0.0024) 0.9827 (0.0142)	0.5021 (0.0024) 0.9855 (0.0145)		0.4948 (0.0024) 0.9721 (0.0146)

Sch.—scheme, Parm.—Parameter.

**Table 12:** Avg. and MSE (in practices) for BEs (MCMC) of the Kum. distribution for different PCS-II when $\alpha =1$ , $\beta =1$ at different values of $n$ and $m$.

$(n,m)$	Sch	Parm	BEs: MCMC				
			SE	LINEX		GE	
				$c =-0.5$	$c=1.5$	$q=0.1$	$q=2$
(40, 20)	${S}_{1}$	$\alpha$ $\beta$	0.9755 (0.0145) 0.8764 (0.0336)	0.9832 (0.0146) 0.8819 (0.0328)	0.9533 (0.0150) 0.8607 (0.0363)	0.9585 (0.0152) 0.8633 (0.0364)	0.9287 (0.0179) 0.8406 (0.0420)
	${S}_{2}$	$\alpha$ $\beta$	0.9439 (0.0130) 0.8109 (0.0457)	0.9501 (0.0126) 0.8153 (0.0443)	0.9258 (0.0146) 0.7979 (0.0502)	0.9296 (0.0145) 0.7991 (0.0500)	0.9045 (0.0182) 0.7788 (0.0580)
	${S}_{3}$	$\alpha$ $\beta$	0.9414 (0.0117) 0.7872 (0.0532)	0.9467 (0.0113) 0.7910 (0.0517)	0.9258 (0.0132) 0.7761 (0.0576)	0.9291 (0.0131) 0.7768 (0.0575)	0.9076 (0.0162) 0.7588 (0.0654)
	${S}_{4}$	$\alpha$ $\beta$	0.9628 (0.0128) 0.8658 (0.0336)	0.9695 (0.0127) 0.8726 (0.0323)	0.9433 (0.0138) 0.8463 (0.0378)	0.9477 (0.0139) 0.8493 (0.0377)	0.9207 (0.0168) 0.8209 (0.0459)
(40, 30)	${S}_{1}$	$\alpha$ $\beta$	1.0032 (0.0103) 0.9459 (0.0163)	1.0089 (0.0106) 0.9496 (0.0161)	0.9865 (0.0098) 0.9352 (0.0169)	0.9908 (0.0102) 0.9377 (0.0170)	0.9693 (0.0106) 0.9234 (0.0185)
	${S}_{2}$	$\alpha$ $\beta$	0.9960 (0.0084) 0.9264 (0.0148)	1.0010 (0.0086) 0.9296 (0.0145)	0.9813 (0.0083) 0.9171 (0.0159)	0.9850 (0.0084) 0.9191 (0.0158)	0.9658 (0.0091) 0.9063 (0.0177)
	${S}_{3}$	$\alpha$ $\beta$	0.9886 (0.0081) 0.9170 (0.0163)	0.9933 (0.0081) 0.9202 (0.0159)	0.9747 (0.0081) 0.9077 (0.0175)	0.9782 (0.0082) 0.9096 (0.0174)	0.9599 (0.0091) 0.8967 (0.0196)
	${S}_{4}$	$\alpha$ $\beta$	1.0081 (0.0091) 0.9530 (0.0144)	1.0133 (0.0094) 0.9569 (0.0143)	0.9929 (0.0085) 0.9414 (0.0150)	0.9969 (0.0088) 0.9441 (0.0151)	0.9773 (0.0090) 0.9288 (0.0166)
(80, 40)	${S}_{1}$	$\alpha$ $\beta$	0.9976 (0.0154) 0.9528 (0.0243)	1.0036 (0.0158) 0.9574 (0.0244)	0.9802 (0.0149) 0.9393 (0.0243)	0.9846 (0.0154) 0.9426 (0.0247)	0.9620 (0.0161) 0.9250 (0.0260)
	${S}_{2}$	$\alpha$ $\beta$	0.9721 (0.0117) 0.9166 (0.0237)	0.9770 (0.0116) 0.9215 (0.0234)	0.9576 (0.0122) 0.9026 (0.0251)	0.9610 (0.0123) 0.9055 (0.0252)	0.9417 (0.0138) 0.8864 (0.0283)
	${S}_{3}$	$\alpha$ $\beta$	0.9819 (0.0107) 0.9279 (0.0231)	0.9863 (0.0108) 0.9328 (0.0228)	0.9690 (0.0109) 0.9137 (0.0241)	0.9722 (0.0110) 0.9167 (0.0243)	0.9551 (0.0120) 0.8975 (0.0269)
	${S}_{4}$	$\alpha$ $\beta$	1.0006 (0.0111) 0.9745 (0.0225)	1.0054 (0.0114) 0.9812 (0.0229)	0.9863 (0.0108) 0.9555 (0.0220)	0.9899 (0.0111) 0.9603 (0.0227)	0.9712 (0.0115) 0.9360 (0.0239)
(80, 60)	${S}_{1}$	$\alpha$ $\beta$	1.0100 (0.0117) 0.9873 (0.0151)	1.0142 (0.0120) 0.9903 (0.0152)	0.9976 (0.0111) 0.9786 (0.0148)	1.0009 (0.0114) 0.9809 (0.0150)	0.9851 (0.0114) 0.9697 (0.0152)
	${S}_{2}$	$\alpha$ $\beta$	1.0026 (0.0098) 0.9748 (0.0135)	1.0065 (0.0099) 0.9778 (0.0135)	0.9914 (0.0095) 0.9662 (0.0135)	0.9943 (0.0097) 0.9684 (0.0136)	0.9798 (0.0098) 0.9572 (0.0141)
	${S}_{3}$	$\alpha$ $\beta$	1.0005 (0.0084) 0.9674 (0.0123)	1.0039 (0.0085) 0.9701 (0.0123)	0.9904 (0.0082) 0.9594 (0.0125)	0.9931 (0.0083) 0.9614 (0.0126)	0.9801 (0.0085) 0.9510 (0.0132)
	${S}_{4}$	$\alpha$ $\beta$	1.0128 (0.0100) 0.9921 (0.0147)	1.0166 (0.0103) 0.9955 (0.0149)	1.0016 (0.0095) 0.9821 (0.0143)	1.0046 (0.0098) 0.9848 (0.0146)	0.9903 (0.0096) 0.9721 (0.0146)

Sch.—scheme, Parm.—Parameter.

**Table 13:** Avg. and MSE (in practices) for BEs (MCMC) of the Kum. distribution for different PCS-II when $\alpha =1$ , $\beta =2$ at different values of $n$ and $m$.

$(n,m)$	Sch	Parm	BEs: MCMC				
			SE	LINEX		GE	
				$c =-0.5$	$c=1.5$	$q=0.1$	$q=2$
(40, 20)	${S}_{1}$	$\alpha$ $\beta$	0.9804 (0.0106) 1.8117 (0.1286)	0.9859 (0.0106) 1.8417 (0.1254)	0.9644 (0.0109) 1.7304 (0.1487)	0.9682 (0.0111) 1.7778 (0.1383)	0.9469 (0.0125) 1.7192 (0.1607)
	${S}_{2}$	$\alpha$ $\beta$	0.9548 (0.0090) 1.6830 (0.1541)	0.9592 (0.0087) 1.7089 (0.1418)	0.9417 (0.0100) 1.6127 (0.1951)	0.9446 (0.0099) 1.6512 (0.1731)	0.9268 (0.0119) 1.5964 (0.2107)
	${S}_{3}$	$\alpha$ $\beta$	0.9529 (0.0082) 1.6381 (0.1739)	0.9567 (0.0079) 1.6597 (0.1612)	0.9417 (0.0091) 1.5785 (0.2145)	0.9442 (0.0089) 1.6106 (0.1931)	0.9290 (0.0107) 1.5629 (0.2298)
	${S}_{4}$	$\alpha$ $\beta$	0.9746 (0.0087) 1.7986 (0.1221)	0.9794 (0.0086) 1.8392 (0.1150)	0.9603 (0.0092) 1.6937 (0.1582)	0.9637 (0.0092) 1.7533 (0.1382)	0.9444 (0.0107) 1.6758 (0.1755)
(40, 30)	${S}_{1}$	$\alpha$ $\beta$	1.0040 (0.0072) 1.9320 (0.0719)	1.0079 (0.0073) 1.9519 (0.0727)	0.9924 (0.0069) 1.8760 (0.0745)	0.9954 (0.0071) 1.9103 (0.0735)	0.9805 (0.0073) 1.8729 (0.0785)
	${S}_{2}$	$\alpha$ $\beta$	0.9977 (0.0056) 1.8941 (0.0599)	1.0011 (0.0057) 1.9118 (0.0585)	0.9875 (0.0056) 1.8440 (0.0679)	0.9901 (0.0057) 1.8743 (0.0634)	0.9770 (0.0060) 1.8401 (0.0712)
	${S}_{3}$	$\alpha$ $\beta$	0.9908 (0.0055) 1.8702 (0.0643)	0.9940 (0.0056) 1.8875 (0.0621)	0.9812 (0.0056) 1.8214 (0.0743)	0.9836 (0.0057) 1.8507 (0.0687)	0.9711 (0.0061) 1.8169 (0.0779)
	${S}_{4}$	$\alpha$ $\beta$	1.0089 (0.0061) 1.9489 (0.0630)	1.0125 (0.0062) 1.9709 (0.0641)	0.9984 (0.0058) 1.8874 (0.0655)	1.0012 (0.0059) 1.9251 (0.0643)	0.9877 (0.0059) 1.8841 (0.0694)
(80, 40)	${S}_{1}$	$\alpha$ $\beta$	1.0006 (0.0113) 1.9510 (0.1146)	1.0048 (0.0115) 1.9772 (0.1202)	0.9880 (0.0110) 1.8793 (0.1084)	0.9913 (0.0113) 1.9236 (0.1138)	0.9750 (0.0116) 1.8765 (0.1161)
	${S}_{2}$	$\alpha$ $\beta$	0.9789 (0.0084) 1.8756 (0.1028)	0.9825 (0.0084) 1.9042 (0.1028)	0.9682 (0.0087) 1.7985 (0.1131)	0.9708 (0.0087) 1.8445 (0.1080)	0.9567 (0.0096) 1.7914 (0.1214)
	${S}_{3}$	$\alpha$ $\beta$	0.9903 (0.0077) 1.9101 (0.1025)	0.9934 (0.0077) 1.9380 (0.1049)	0.9810 (0.0077) 1.8341 (0.1059)	0.9833 (0.0078) 1.8802 (0.1050)	0.9711 (0.0082) 1.8287 (0.1137)
	${S}_{4}$	$\alpha$ $\beta$	1.0042 (0.0078) 1.9955 (0.1082)	1.0077 (0.0080) 2.0346 (0.1189)	0.9937 (0.0076) 1.8934 (0.0980)	0.9964 (0.0078) 1.9562 (0.1052)	0.9828 (0.0080) 1.8893 (0.1074)
(80, 60)	${S}_{1}$	$\alpha$ $\beta$	1.0094 (0.0082) 2.0025 (0.0746)	1.0124 (0.0084) 2.0189 (0.0779)	1.0008 (0.0079) 1.9560 (0.0688)	1.0031 (0.0081) 1.9853 (0.0731)	0.9920 (0.0080) 1.9557 (0.0720)
	${S}_{2}$	$\alpha$ $\beta$	1.0037 (0.0068) 1.9798 (0.0658)	1.0064 (0.0068) 1.9966 (0.0680)	0.9958 (0.0066) 1.9324 (0.0631)	0.9979 (0.0067) 1.9620 (0.0653)	0.9876 (0.0068) 1.9314 (0.0660)
	${S}_{3}$	$\alpha$ $\beta$	1.0025 (0.0058) 1.9671 (0.0589)	1.0049 (0.0059) 1.9822 (0.0602)	0.9955 (0.0057) 1.9242 (0.0580)	0.9973 (0.0058) 1.9510 (0.0590)	0.9884 (0.0058) 1.9230 (0.0604)
	${S}_{4}$	$\alpha$ $\beta$	1.0134 (0.0069) 2.0186 (0.0736)	1.0160 (0.0070) 2.0382 (0.0780)	1.0055 (0.0066) 1.9640 (0.0660)	1.0076 (0.0067) 1.9984 (0.0714)	0.9976 (0.0066) 1.9637 (0.0695)

**Table 14:** HPD-CI, AILs and CPs (in %) for BEs (MCMC) of the Kum. distribution for different PCS-II when $\alpha =0.5$ at different values of $n$ and $m$.

$(n,m)$	Sch	Parm	HPD CI $\alpha =0.5$ ,$\beta =0.5$		HPD CI $\alpha =0.5$ ,$\beta =1$	
			(Lower, Upper)	AIL/CP	(Lower, Upper)	AIL/CP
(40, 20)	${S}_{1}$	$\alpha$ $\beta$	(0.3565, 0.6367) (0.3160, 0.5549)	0.2801/98.2 0.2388/97.1	(0.3735, 0.6048) (0.6270, 1.1527)	0.2312/97.6 0.5257/96.6
	${S}_{2}$	$\alpha$ $\beta$	(0.3496, 0.5903) (0.3101, 0.4843)	0.2406/97.4 0.1742/97.7	(0.3716, 0.5677) (0.6218, 1.0131)	0.1961/97.0 0.3912/97.1
	${S}_{3}$	$\alpha$ $\beta$	(0.3521, 0.5738) (0.3047, 0.4591)	0.2216/97.1 0.1544/97.2	(0.3926, 0.5713) (0.6215, 0.9715)	0.1787/98.9 0.3500/97.6
	${S}_{4}$	$\alpha$ $\beta$	(0.3510, 0.6118) (0.3146, 0.5331)	0.2608/97.2 0.2185/97.1	(0.3762, 0.5874) (0.6054, 1.1021)	0.2111/97.1 0.4967/96.2
(40, 30)	${S}_{1}$	$\alpha$ $\beta$	(0.3758, 0.6224) (0.3622, 0.5677)	0.2465/96.6 0.2054/96.5	(0.4017, 0.5980) (0.7339, 1.1893)	0.1963/97.2 0.4553/97.1
	${S}_{2}$	$\alpha$ $\beta$	(0.3846, 0.6114) (0.3732, 0.5438)	0.2268/97.1 0.1705/97.3	(0.4080, 0.5860) (0.7410, 1.1225)	0.1780/97.1 0.3815/97.1
	${S}_{3}$	$\alpha$ $\beta$	(0.3843, 0.6015) (0.3715, 0.5434)	0.2171/97.0 0.1719/97.6	(0.4052, 0.5792) (0.7331, 1.1128)	0.1740/96.9 0.3797/97.1
	${S}_{4}$	$\alpha$ $\beta$	(0.3857, 0.6192) (0.3743, 0.5733)	0.2334/96.9 0.1989/97.2	(0.4089, 0.5930) (0.7363, 1.1767)	0.1841/96.9 0.4403/96.8
(80, 40)	${S}_{1}$	$\alpha$ $\beta$	(0.3630, 0.6548) (0.3395, 0.5999)	0.2917/97.5 0.2603/96.3	(0.3865, 0.6288) (0.6787, 1.2446)	0.2422/97.7 0.5658/96.3
	${S}_{2}$	$\alpha$ $\beta$	(0.3561, 0.6025) (0.3360, 0.5649)	0.2464/96.4 0.2289/96.3	(0.3801, 0.5849) (0.6787, 1.1662)	0.2047/96.7 0.4875/96.7
	${S}_{3}$	$\alpha$ $\beta$	(0.3705, 0.6154) (0.3365, 0.5680)	0.2449/97.3 0.2314/96.3	(0.3941, 0.5929) (0.6926, 1.2019)	0.1988/97.2 0.5093/97.0
	${S}_{4}$	$\alpha$ $\beta$	(0.3685, 0.6234) (0.3577, 0.6111)	0.2549/96.5 0.2533/96.8	(0.4060, 0.6137) (0.7048, 1.2537)	0.2076/98.1 0.5489/96.6
(80, 60)	${S}_{1}$	$\alpha$ $\beta$	(0.3755, 0.6347) (0.3855, 0.5955)	0.2592/96.4 0.2099/96.6	(0.4026, 0.6119) (0.7573, 1.2206)	0.2093/97.2 0.4632/96.2
	${S}_{2}$	$\alpha$ $\beta$	(0.3787, 0.6200) (0.3871, 0.5810)	0.2413/96.3 0.1939/96.8	(0.4067, 0.5979) (0.7599, 1.1952)	0.1911/97.2 0.4352/96.3
	${S}_{3}$	$\alpha$ $\beta$	(0.3855, 0.6101) (0.3890, 0.5702)	0.2245/96.4 0.1811/96.9	(0.4106, 0.5878) (0.7686, 1.1767)	0.1771/97.2 0.4080/96.5
	${S}_{4}$	$\alpha$ $\beta$	(0.3852, 0.6271) (0.3869, 0.5972)	0.2418/96.5 0.2103/96.6	(0.4132, 0.6045) (0.7659, 1.2308)	0.1913/97.4 0.4648/96.4

Sch.—scheme, Parm.—Parameter.

**Table 15:** HPD-CI, AILs and CPs (in %) for BEs (MCMC) of the Kum. distribution for different PCS-II when $\alpha =1$ at different values of $n$ and $m$.

$(n,m)$	Sch	Parm	HPD CI $\alpha =1$ ,$\beta =1$		HPD CI $\alpha =1$ ,$\beta =2$	
			(Lower, Upper)	AIL/CP	(Lower, Upper)	AIL/CP
(40, 20)	${S}_{1}$	$\alpha$ $\beta$	(0.7565, 1.2182) (0.6159, 1.1426)	0.4617/97.9 0.5267/96.2	(0.7919, 1.1885) (1.2583, 2.4322)	0.3966/98.0 1.1738/96.8
	${S}_{2}$	$\alpha$ $\beta$	(0.7448, 1.1386) (0.6212, 1.0095)	0.3938/97.0 0.3882/97.2	(0.7804, 1.1097) (1.2406, 2.1550)	0.3292/96.7 0.9143/97.2
	${S}_{3}$	$\alpha$ $\beta$	(0.7478, 1.1058) (0.6125, 0.9619)	0.3579/96.4 0.3494/97.0	(0.7947, 1.0977) (1.2401, 2.0539)	0.3030/97.0 0.8137/97.1
	${S}_{4}$	$\alpha$ $\beta$	(0.7553, 1.1761) (0.6010, 1.0912)	0.4207/97.1 0.4902/96.0	(0.7972, 1.1528) (1.1885, 2.3195)	0.3555/97.2 1.1309/95.9
(40, 30)	${S}_{1}$	$\alpha$ $\beta$	(0.8015, 1.1997) (0.7320, 1.1854)	0.3982/97.1 0.4534/97.0	(0.8318, 1.1651) (1.4359, 2.4520)	0.3332/96.9 1.0160/96.5
	${S}_{2}$	$\alpha$ $\beta$	(0.8142, 1.1728) (0.7446, 1.1279)	0.3586/97.1 0.3832/97.3	(0.8480, 1.1427) (1.4605, 2.3329)	0.2946/97.3 0.8723/96.7
	${S}_{3}$	$\alpha$ $\beta$	(0.8112, 1.1614) (0.7333, 1.1125)	0.3501/97.0 0.3791/97.1	(0.8365, 1.1271) (1.4437, 2.3043)	0.2906/96.3 0.8606/96.8
	${S}_{4}$	$\alpha$ $\beta$	(0.8170, 1.1873) (0.7484, 1.1876)	0.3703/96.9 0.4391/97.2	(0.8533, 1.1583) (1.4816, 2.4568)	0.3049/97.2 0.9752/97.0
(80, 40)	${S}_{1}$	$\alpha$ $\beta$	(0.7454, 1.2305) (0.6733, 1.2365)	0.4850/96.2 0.5631/96.1	(0.7753, 1.1940) (1.3694, 2.6097)	0.4187/96.1 1.2402/96.7
	${S}_{2}$	$\alpha$ $\beta$	(0.7464, 1.1540) (0.6786, 1.1642)	0.4076/95.9 0.4855/96.7	(0.8136, 1.1633) (1.3354, 2.4379)	0.3497/97.8 1.1024/96.5
	${S}_{3}$	$\alpha$ $\beta$	(0.7787, 1.1791) (0.6898, 1.1998)	0.4003/96.5 0.5099/96.9	(0.8213, 1.1600) (1.3601, 2.5315)	0.3387/97.1 1.1713/96.7
	${S}_{4}$	$\alpha$ $\beta$	(0.8109, 1.2223) (0.7057, 1.2563)	0.4114/97.9 0.5506/96.7	(0.8325, 1.1790) (1.3717, 2.6001)	0.3464/97.4 1.2283/96.1
(80, 60)	${S}_{1}$	$\alpha$ $\beta$	(0.8125, 1.2317) (0.7537, 1.2176)	0.4191/97.6 0.4639/96.1	(0.8377, 1.1898) (1.5286, 2.5863)	0.3521/97.4 1.0577/97.1
	${S}_{2}$	$\alpha$ $\beta$	(0.8184, 1.2003) (0.7566, 1.1928)	0.3819/97.7 0.4362/96.3	(0.8473, 1.1633) (1.5093, 2.5015)	0.3159/97.6 0.9921/96.6
	${S}_{3}$	$\alpha$ $\beta$	(0.8173, 1.1744) (0.7641, 1.1738)	0.3571/96.9 0.4096/96.4	(0.8733, 1.1689) (1.5402, 2.4812)	0.2956/99.1 0.9409/97.4
	${S}_{4}$	$\alpha$ $\beta$	(0.8178, 1.2025) (0.7648, 1.2301)	0.3847/96.9 0.4652/96.3	(0.8665, 1.1830) (1.5173, 2.5728)	0.3165/98.4 1.0554/96.6

Sch.—scheme, Parm.—Parameter.

Based on Tables 2, 3, 4, 5, 6, 7, 8, 9, 10, 11, 12, 13, with an increase in the values of $(n, m)$, and specifically an increase in the value of $m$ leads to a decrease in the MSEs, in addition to that the Avg. denotes the true value of the two parameters $\alpha$ and $\beta$, for all estimation methods. By comparing the performance of Non-BE methods, we note that estimates of MLE are more efficient than estimates of MPS. In comparison between the performance of the BE methods relative to the Lindley’s approximation, we find that the value of the MSEs decreases under the LINEX loss function when $c = -0.5$, then followed by the SE loss function. As for the MCMC algorithm, we find that the MSEs value decreases under the SE loss function. In general, we note that estimates of MCMC are more efficient than estimates of Lindley’s approximation. From Tables 14, 15, it is observed that the HPD and Asy CI have the smallest and largest average lengths, respectively. As a general result, we see that when $n$ increases, for all cases, the AIL decrease and the corresponding CP percentages increase.

**Figure 2:**
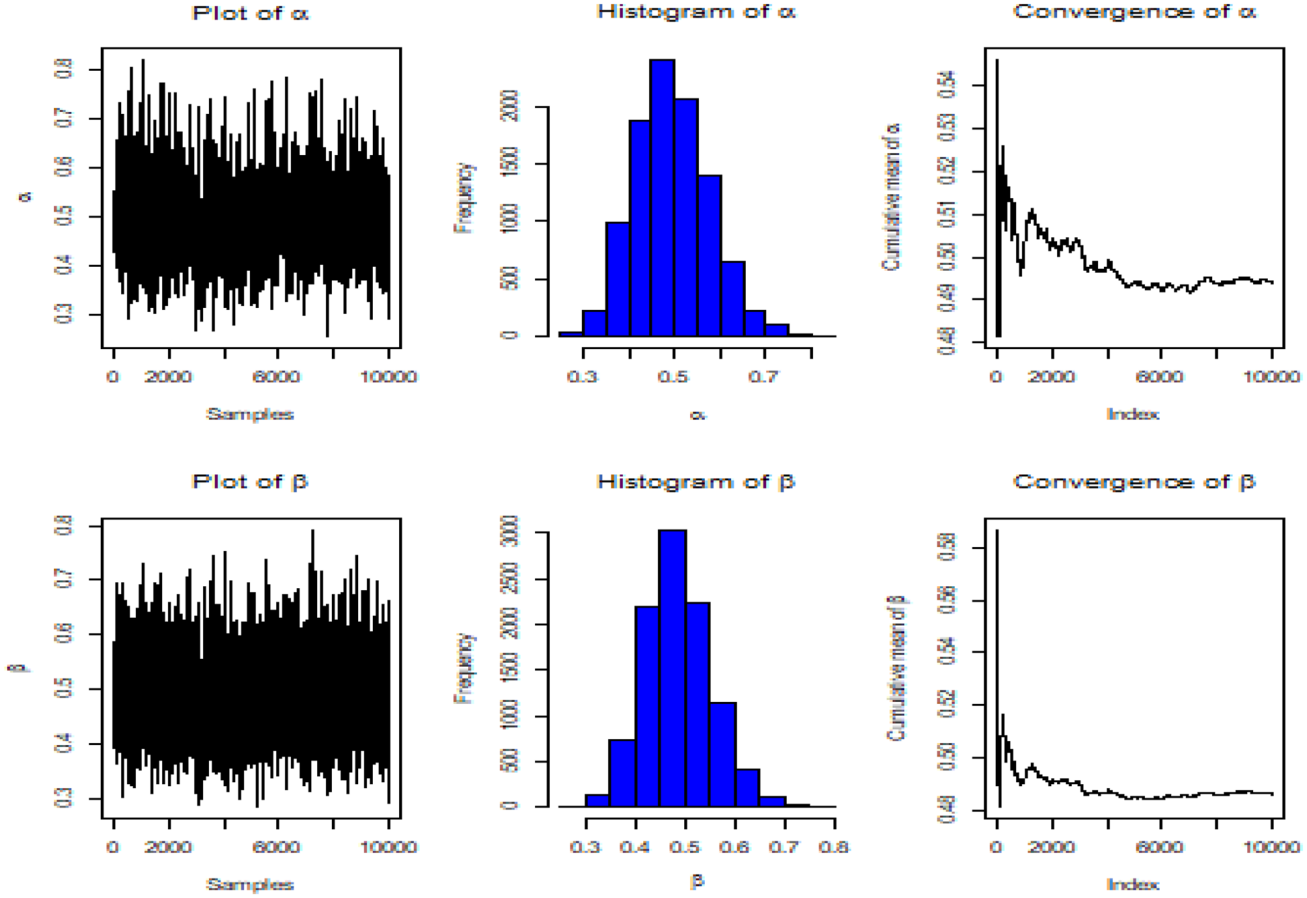
Convergence of MCMC Estimates.

The graphs of MCMC estimates for $\alpha$ and $\beta$ using MH algorithm are the plotting of estimates, histogram of estimates, and convergence of estimates, these graphs can be showed in Fig. 2. In Fig. 2 the plots display the random distribution of the values of $\alpha$ and $\beta$, which are observed to be scattered around the mean. Also, from the histograms of the MH sequences for $\alpha$, we observe that choosing the normal distribution as a proposal distribution is quite appropriate.

### Real data application

In this subsection, we examine actual data that pertains to the Shasta reservoir's monthly water capacity in California, USA. The data covers the month of February between 1991 and 2010. see Sultana et al.^25^ and Sultana et al.^12^. The data points are listed below as follows.

$$ \begin{aligned} & 0.338936, \, 0.431915, \, 0.759932, \, 0.724626, \, 0.757583, \, 0.811556, \, 0.785339, \\ & 0.783660, \, 0.815627, \, 0.847413, 0.768007, \, 0.843485, \, 0.787408, \, 0.849868, \\ & 0.695970, \, 0.842316, \, 0.828689, 0.580194, 0.430681, 0.742563. \\ \end{aligned} $$

**Figure 3:**
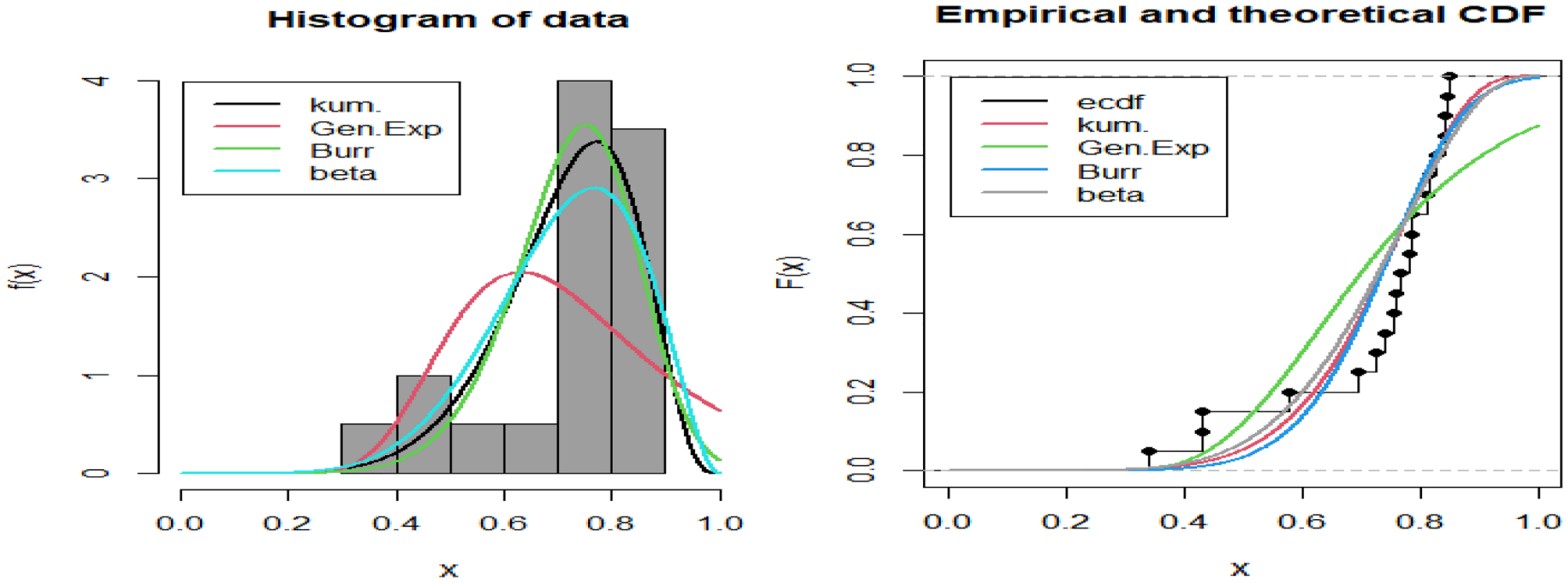
Goodness of Fit Tests for Real Data.

To determine if the considered dataset can be appropriately analyzed using a Kum. distribution, a goodness of fit test is conducted. In addition to Kum. distribution, we also fit generalized exponential [Gen.Exp], Burr XII [Burr], and beta distributions to the data set. We judge the goodness of fit using various criteria, for example, negative log-likelihood criterion (NLC), Akaike information criterion (AIC) introduced by Akaike^26^, corrected AIC (AICc) introduced by Hurvich and Tsai^27^, and Bayesian information criterion (BIC) introduced by Schwarz^28^. The smaller the value of these criteria, the better the model fits the data. The results are shown in Table 16. For fitting the given data set graphically, the empirical cdf can be plotted with the corresponding fitted cdfs for Kum., Gen.Exp, Burr, and beta distributions. Also, the histogram can be plotted with the corresponding fitted pdf lines for the same distributions. Figure 3 showed the fitted lines for the cdfs and pdfs for the given data set and corresponding distributions. The figures also indicate that the Kum. distribution provide better fit than the other distributions at least for this data set.

**Table 16:** Goodness of fit tests for different distributions for real data.

Distribution	NLS	AIC	AICc	BIC	K-S	P-value
Kum	−13.4747	−22.9494	−22.2435	−20.9579	0.2208	0.2446
Gen.Exp	−4.7925	−5.5851	−4.8791	−3.5936	0.2948	0.0490
Burr	−11.5059	−19.0118	−18.3059	−17.0204	0.2247	0.2276
beta	−12.5619	−21.1238	−20.4179	−19.1323	0.2359	0.1833

Referring to the values reported in Table 16, we conclude that the Kum. distribution fits the data set good compared to the other models. Thus, the various point and interval estimates of $\alpha$ and $\beta$ for real data under PCS-II as following as in Tables 17, 18, 19.

**Table 17:** Point and interval estimates of $\alpha$ and $\beta$ for Non-BEs of the Kum. distribution under PCS-II.

Sch	Parm	Non-Bayesian		Asy CI
		MLE	MPS	
${S}_{1}$	$\alpha$ $\beta$	7.0290 (2.2955) 5.5579 (3.7248)	12.9105 (4.3516) 23.4821 (24.7160)	(2.5297, 11.5282) (0.0000, 12.8584)
${S}_{2}$	$\alpha$ $\beta$	5.5999 (2.0037) 1.9433 (1.1408)	8.6988 (3.2672) 3.7516 (2.9224)	(1.6727, 9.5272) (0.0000, 4.1793)
${S}_{3}$	$\alpha$ $\beta$	8.1085 (2.7864) 3.8957 (3.1207)	17.2251 (5.2833) 36.6941 (47.8674)	(2.6472, 13.5698) (0.0000, 10.0123)
${S}_{4}$	$\alpha$ $\beta$	5.1171 (1.9620) 1.1644 (0.6658)	7.8385 (3.0958) 2.0032 (1.4750)	(1.2716, 8.9626) (0.0000, 2.4695)

**Table 18:** Point and interval estimates of $\alpha$ and $\beta$ for BEs (Lindely) of the Kum. distribution under PCS-II.

Sch	Parm	BEs: Lindely				
		SE	LINEX		GE	
			$c =-0.5$	$c=1.5$	$q=0.1$	$q=2$
${S}_{1}$	$\alpha$ $\beta$	7.6170 4.5491	7.7592 5.3061	4.9488 3.1666	7.5450 4.3994	6.2426 3.8111
${S}_{2}$	$\alpha$ $\beta$	6.1793 1.5877	6.3227 1.6634	5.3561 1.4953	6.0868 1.5360	5.8881 1.5056
${S}_{3}$	$\alpha$ $\beta$	8.6793 3.2333	8.8331 3.5905	7.8360 3.0233	8.6138 3.1184	8.4795 3.0419
${S}_{4}$	$\alpha$ $\beta$	5.6805 0.9580	5.8308 0.9840	4.8560 0.9111	5.5752 0.9255	5.3504 0.9052

**Table 19:** Point and interval estimates of $\alpha$ and $\beta$ for BEs (MCMC) of the Kum. distribution under PCS-II.

Sch	Parm	BEs: MCMC					HPD CI
		SE	LINEX		GE		
			$c =-0.5$	$c=1.5$	$q=0.1$	$q=2$	
${S}_{1}$	$\alpha$ $\beta$	7.1066 (1.7113) 5.8185 (2.7249)	7.7237 8.3947	4.9488 3.1666	6.8371 5.0892	6.2426 3.8111	(3.6423, 10.0769) (1.2250, 10.8947)
${S}_{2}$	$\alpha$ $\beta$	8.5666 (2.1150) 5.3924 (2.7475)	9.5128 7.6374	6.5988 3.0663	8.2638 4.6392	7.7440 3.5589	(5.5505, 11.5230) (1.5973, 10.6698)
${S}_{3}$	$\alpha$ $\beta$	13.3481 (3.6459) 23.2557 (17.1281)	15.6215 80.1046	9.2612 5.1644	12.7577 15.8284	11.7370 8.4052	(7.9778, 18.1030) (2.9379, 53.5559)
${S}_{4}$	$\alpha$ $\beta$	6.0220 (0.9607) 2.0273 (0.6169)	6.2433 2.1285	5.4002 1.7887	5.9350 1.9258	5.7805 1.7520	(4.4033, 7.4454) (0.8945, 3.2152)

In Table 17, we display the Non-BE obtained by using MLE and MPS at $m=10$. We computed the average estimates (Avg.), standard deviation, and the asymptotic confidence interval (Asy CI) using the MLE.

In Tables 18, 19, we display the BE obtained by using Lindley’s approximation and MCMC method under different loss functions at $m=10$ with different four schemes defined as: ${S}_{1}=\left(10, {0}^{*9}\right), {S}_{2}=\left(5,{0}^{*8}, 5\right),{S}_{3}=\left({0}^{*4}, \mathrm{5,5},{0}^{*4}\right), \;\mathrm{and}\;{S}_{4}=( {0}^{*9}, 10)$. We computed the average estimates (Avg.), standard deviation, and the highest posterior density (HPD) intervals using the MCMC. From Tables 17, 18, 19, we observed that the Avg. and MSEs of the different estimates are close together. MPS have the worst performance and Bayesian estimates have the best performance.

**Figure 4:**
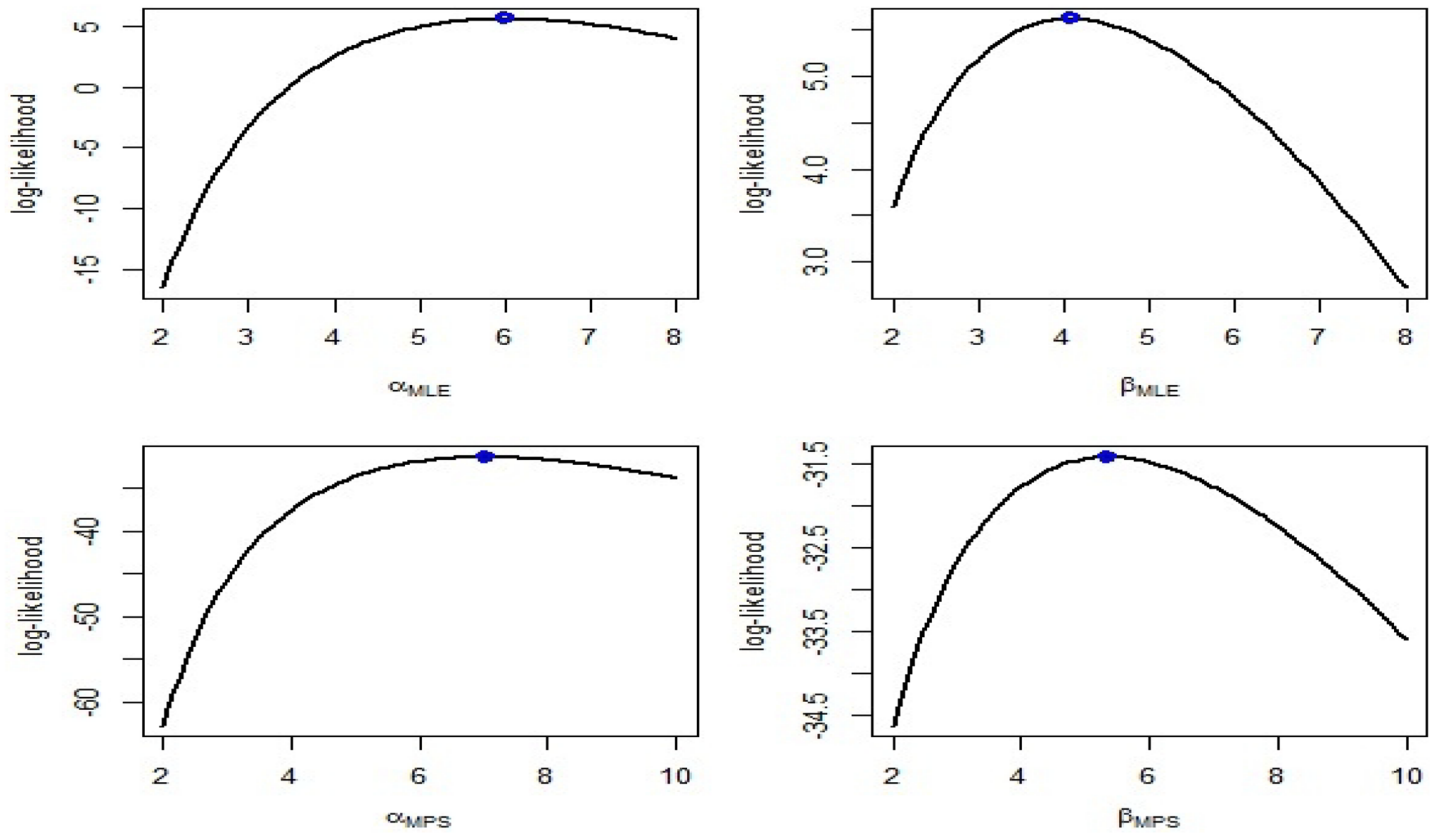
The Profile-likelihood of The Estimates of MLE and MPS Under PCS-II.

Figure 4 showed the profile-likelihood of the estimates of MLE and MPS under PCS-II: ${S}_{1}=\left(10, {0}^{*9}\right)$ with $m =10$ for the parameters $\alpha$ and $\beta$. Form the Fig. 4, we can conclude the existences and uniqueness for the estimates of MLE and MPS where the maximum value of likelihood functions of MLE and MPS given estimates of $\alpha$ and $\beta$ has been achieved.

## Optimal progressive Type-II censoring scheme

In the preceding sections, we have discussed both Non-BE and BE methods for estimating unknown parameters of the Kum. distribution when using PCS-II to obtain samples. To conduct a life-testing experiment using PCS-II, it is necessary to have advance knowledge of $n, m,$ and $({R}_{1},{R}_{2}, \dots ,{R}_{m})$. However, in many reliability and life testing studies, practical considerations require selecting the optimal PCS-II from a set of possible schemes. Balakrishnan and Aggarwala^5^ extensively discussed the problem of determining the best censoring plan using different setups. Comparing two different censoring schemes has been of great interest to several researchers. See, for example, Ng et al.^29^, Kundu^30^^,^ Lee et al.^31^, Lee et al.^32^, and Ashour et al.^33^. To determine the optimum PCS-II, we consider an information measure as the following criteria.

**Criterion 1:** Minimizing the determinant of the variance–covariance matrix $({I}^{-1})$ of the MLEs has been obtained in Eq. (6).

$$det\left[{I}^{-1}\right]=var\left(\widehat{\alpha }\right)var\left(\widehat{\beta }\right)-{\left(cov\left(\widehat{\alpha },\widehat{\beta }\right)\right)}^{2},$$

**Criterion 2:** Minimizing the trace of the variance–covariance matrix $({I}^{-1})$ of the MLEs has been obtained in Eq. (6).

$$tr\left[{I}^{-1}\right]=var\left(\widehat{\alpha }\right)+var\left(\widehat{\beta }\right),$$

**Criterion 3:** depends on the choice of $u$, and it is tends to minimize the variance of logarithmic of MLE of the ${u}^{-th}$ quantile $\left(\mathrm{log}\left({\widehat{T}}_{u}\right)\right)$, where $0<u<1$. Consider the ${u}^{-th}$ quantile of the Kum. distribution:

$${T}_{u} = {\left({1-\left(1-u\right)}^{\frac{1}{\beta }}\right)}^{\frac{1}{\alpha }}$$

Hence, the logarithmic for ${T}_{u}$ of the Kum. distribution is given by

13 $$\mathrm{log}\left({T}_{u}\right)=\frac{1}{\alpha }\mathrm{log}\left({1-\left(1-u\right)}^{\frac{1}{\beta }}\right), 0<u<1$$

From Eq. (13), using the delta method, the variance of $\mathrm{log}\left({\widehat{T}}_{u}\right)$ can be approximated by

$$Var\left(\mathrm{log}\left({\widehat{T}}_{u}\right)\right)={\left[\nabla \mathrm{log}\left({\widehat{T}}_{u}\right)\right]}^{T}{I}^{-1}\left[\nabla \mathrm{log}\left({\widehat{T}}_{u}\right)\right]$$

where

$${\left[\nabla \mathrm{log}\left({\widehat{T}}_{u}\right)\right]}^{T}= {\left[\frac{\partial \nabla \mathrm{log}\left({T}_{u}\right)}{\partial \alpha }, \frac{\partial \nabla \mathrm{log}\left({T}_{u}\right)}{\partial \beta } \right]}_{\alpha =\widehat{\alpha }, \beta =\widehat{\beta }}$$

is the gradient of $\mathrm{log}\left({T}_{u}\right)$ with respect to the unknown parameters $\alpha$ and $\beta$.where

$$ \begin{aligned} \frac{{\partial \nabla \log \left( {T_{u} } \right)}}{\partial \alpha } & = \frac{ - 1}{{\alpha^{2} }}\log \left( {1 - \left( {1 - u} \right)^{{\frac{1}{\beta }}} } \right), \\ \frac{{\partial \nabla \log \left( {T_{u} } \right)}}{\partial \beta } & = \frac{1}{\alpha }\left[ {\frac{{\frac{1}{{\beta^{2} }}\left( {1 - u} \right)^{{\frac{1}{\beta }}} \log \left( {1 - u} \right)}}{{1 - \left( {1 - u} \right)^{{\frac{1}{\beta }}} }}} \right] \\ \end{aligned} $$

Thus, the variance of $\mathrm{log}\left({\widehat{T}}_{u}\right)$ can be obtained as

$$Var\left(\mathrm{log}\left({\widehat{T}}_{u}\right)\right)=\left[\frac{\partial \nabla \mathrm{log}\left({T}_{u}\right)}{\partial \alpha } \frac{\partial \nabla \mathrm{log}\left({T}_{u}\right)}{\partial \beta } \right]\left[\begin{array}{cc}var(\widehat{\alpha })& cov(\widehat{\alpha },\widehat{\beta })\\ cov(\widehat{\beta },\widehat{\alpha })& var(\widehat{\beta })\end{array}\right]\left[\begin{array}{c}\frac{\partial \nabla \mathrm{log}\left({T}_{u}\right)}{\partial \alpha }\\ \frac{\partial \nabla \mathrm{log}\left({T}_{u}\right)}{\partial \beta }\end{array}\right]$$

The calculated values of determinant and trace of the variance–covariance matrix of the MLEs when $\alpha =0.5$, $\beta =1, n=\left(\mathrm{40,80}\right) \mathrm{and m}=(\mathrm{20,30,40},\mathrm{ and }60)$ are presented in Table 20. Based on three different quantiles namely: $u=0.25, 0.5\mathrm{ and }0.75$, the Criterion 3 is computed. The calculated values of the three criteria are reported in Table 20.

**Table 20:** Optimal censoring scheme of the Kum. distribution for different Criteria when $\alpha =0.5$ , $\beta =1$ at different values of $n$ and $m$.

$(n, m)$	Sch	Criterion 1	Criterion 2	Criterion 3		
				$u=0.25$	$u=0.5$	$u=0.75$
(40, 20)	${S}_{1}=(20, {0}^{*19})$	0.0015	0.1570	0.3173	0.1101	0.0452
	${S}_{2}=(10,{0}^{*18}, 10)$	0.0023	0.2836	0.2385	0.0953	0.0500
	${S}_{3}=({0}^{*9}, \mathrm{10,10},{0}^{*9})$	0.0017	0.2505	0.2127	0.0949	0.0506
	${S}_{4}=( {0}^{*19}, 20)$	0.0033	0.4877	0.1950	0.0947	0.0595
(40, 30)	${S}_{1}=(10, {0}^{*29})$	0.0007	0.0952	0.2320	0.0740	0.0286
	${S}_{2}=(5,{0}^{*28}, 5)$	0.0008	0.1135	0.2067	0.0681	0.0291
	${S}_{3}=({0}^{*14}, \mathrm{5,5},{0}^{*14})$	0.0007	0.1106	0.1917	0.0665	0.0294
	${S}_{4}=( {0}^{*29}, 10)$	0.0008	0.1342	0.1867	0.0644	0.0302
(80, 40)	${S}_{1}=(40, {0}^{*39})$	0.0002	0.0604	0.1635	0.0550	0.0218
	${S}_{2}=(20,{0}^{*38}, 20)$	0.0003	0.0884	0.1202	0.0475	0.0244
	${S}_{3}=({0}^{*19}, \mathrm{20,20},{0}^{*19})$	0.0002	0.0793	0.1056	0.0473	0.0248
	${S}_{4}=( {0}^{*39}, 40)$	0.0003	0.1223	0.0973	0.0478	0.0294
(80, 60)	${S}_{1}=(20, {0}^{*59})$	0.0001	0.0401	0.1201	0.0377	0.0142
	${S}_{2}=(10,{0}^{*58}, 10)$	0.0001	0.0456	0.1061	0.0347	0.0145
	${S}_{3}=({0}^{*29}, \mathrm{10,10},{0}^{*29})$	0.0001	0.0445	0.0973	0.0338	0.0147
	${S}_{4}=( {0}^{*59}, 20)$	0.0001	0.0514	0.0952	0.0328	0.0152

Using the previous application in “Real data application” section, we considered a PCS-II sample of size $m$ from the Kum. distribution. Using the variance–covariance matrix of the MLEs, we can easily compute the values of the trace and determinant of the variance–covariance matrix for all choices of $n, m,$ and schemes $({S}_{1},{S}_{2}, \dots ,{S}_{m})$ are presented in Table 21. Based on three different quantiles namely: $u=0.25, 0.5\mathrm{ and }0.75$, the Criterion 3 is computed. These values indicate that the optimal censoring scheme is the one that yields the smallest determinant or trace of the variance–covariance matrix of the MLEs. From Table 21, therefore, the optimum scheme in Criterion 1, 2 is $(n, m, ({S}_{1},{S}_{2}, \dots ,{S}_{m}))= (20, 10, \left( {0}^{*9}, 10\right))$ but in Criterion 3 when the values of $(u=0.25, 0.5)$ the optimum scheme is $(n, m, ({S}_{1},{S}_{2}, \dots ,{S}_{m}))= (20, 10, \left({0}^{*4}, \mathrm{5,5},{0}^{*4}\right))$, and when the value of $u$ was increased, it became the optimum scheme is $(n, m, ({S}_{1},{S}_{2}, \dots ,{S}_{m}))= (20, 10, \left( {0}^{*9}, 10\right))$.

**Table 21:** Comparison of different censoring scheme for application.

$(n, m)$	Censoring schemes	Criterion 1	Criterion 2	Criterion 3		
				$u=0.25$	$u=0.5$	$u=0.75$
$(20, 10)$	${S}_{1}=(10, {0}^{*9})$	16.2781	19.1438	0.0051	0.0022	0.0015
	${S}_{2}=(5,{0}^{*8}, 5)$	1.5162	5.3164	0.0047	0.0022	0.0018
	${S}_{3}=({0}^{*4}, \mathrm{5,5},{0}^{*4})$	11.7832	17.5033	0.0020	0.0013	0.0016
	${S}_{4}=( {0}^{*9}, 10)$	0.5220	4.2929	0.0039	0.0021	0.0014

## Conclusion

This paper deals with the problem of estimating unknown parameters for a Kum. distribution under PCS-II from both BE and Non-BE perspectives. We obtained MLE, MPS, and asymptotic confidence interval estimates for the unknown parameters of a Kum. distribution. We also computed BE, including Lindley’s approximation and MCMC under both symmetric and asymmetric loss functions, along with their corresponding HPD interval estimates. We discussed how to choose hyper-parameter values based on past samples and compared the methods using MSE, AIL, and CP. Our results indicate that BE is superior to non-Bayesian estimates. We identified the optimal censoring scheme for life testing experiments based on three criteria measures, which is important information for reliability practitioners. The future work can be extended to studying neutrosophic statistics for the Kum. distribution. Another work could involve modeling COVID-19 data under different progressive censoring schemes.

## Data Availability

The data is available in this article.
